# Integrating WHO’s digital adaptation kit for antenatal care into BornFyne-PNMS: insights from Cameroon

**DOI:** 10.3389/fphar.2025.1474999

**Published:** 2025-03-26

**Authors:** Miriam Nkangu, Brice Tangang, Arthur Pessa, Donald Weledji, Pamela Obegu, Mwenya Kasonde, Ngo V. Ngo, Franck Wanda, Ronald M. Gobina, Odette Kibu, Veronica Shiroya, Denis Foretia, Choolwe Jacobs, Armel Tassegning, Arone Wondwossen Fantaye, Fobellah Nkengfac, Rosemary K. Muliokela, Tigest Tamrat, Natschja Ratanaprayul, Alice Tabebot, Sanni Yaya

**Affiliations:** ^1^ School of Epidemiology and Public Health, University of Ottawa, Ottawa, ON, Canada; ^2^ Bruyere Health Research Institute, Ottawa, ON, Canada; ^3^ Health Promotion Alliance Cameroon (HPAC), Yaounde, Cameroon; ^4^ Donwel Systems, Brussels, Belgium; ^5^ Liverpool School of Tropical Medicine, Liverpool, United Kingdom; ^6^ Nkafu Policy Institute of the Denis and Lenora Foretia Foundation Cameroon, Yaounde, Cameroon; ^7^ The International Center for Research and Care (CIRES), Akonolinga, Cameroon; ^8^ Centre for Prevention and Digital Health, Medical Faculty Mannheim of Heidelberg University, Heidelberg, Germany; ^9^ Heidelberg Institute of Global Health, Heidelberg University Hospital, Heidelberg, Germany; ^10^ Alliance for Health Promotion, Geneva, Switzerland; ^11^ School of Epidemiology and Public Health University of Zambia, Lusaka, Zambia; ^12^ Faculty of Medicine, University of Ottawa, Ottawa, ON, Canada; ^13^ Ministry of Public Health Cameroon, Yaounde, Cameroon; ^14^ UNDP/UNFPA/UNICEF/WHO/World Bank Special Programme of Research, Development and Research Training in Human Reproduction (HRP), Department of Sexual and Reproductive Health and Research, World Health Organization, Geneva, Switzerland; ^15^ Department of Digital Health and Innovation, World Health Organization, Geneva, Switzerland; ^16^ The George Institute for Global Health, Imperial College London, London, United Kingdom

**Keywords:** digital adaptation kit, SMART guidelines, antenatal care, reproductive maternal newborn child and adolescent health, digital health

## Abstract

**Background:**

Digital health innovations represent unique opportunities to address maternal, newborn, and child health challenges in Sub-Saharan Africa. In 2021, the World Health Organization (WHO) launched the Digital Adaptation Kits (DAKs) for antenatal care (ANC) as part of its Standards-Based, Machine-Readable, Adaptive, Requirements-Based, and Testable (SMART) guidelines approach. DAKs are operational and software-neutral mechanisms that convert WHO guidelines into standardized formats that can be easily integrated into digital systems by various countries. This article outlines the methodology for updating and integrating WHO DAK content into the BornFyne-prenatal management system (PNMS) version 2.0.

**Methods:**

This study, which employs a participatory action research approach, is part of a larger research study for the BornFyne-PNMS project. A review of the ANC DAK operational document and data dictionaries was conducted to identify elements that were present in BornFyne-PNMS version 1.0. This was followed by a series of consultations and stakeholder meetings.

**Results:**

Five stakeholder meetings were held to engage stakeholders across Cameroon. Some of the registration elements, among other DAK aspects of ANC service provision, were identified in BornFyne version 1.0 but required reorganizing, remodeling, and reanalyzing to align with the International Classification of Diseases codes and DAK data content as part of the expansion for BornFyne version 2.0. Up to 40% of the DAK dictionary data content existed within the BornFyne-PNMS version 1.0, including additional DAK content adapted to update BornFyne-PNMS version 2.0. The digital health ecosystem in Cameroon is in an emerging phase with an increasing demand for digital health technologies, especially in the areas of reproductive, maternal, newborn, child, and adolescent health.

**Conclusion:**

The digital health ecosystem in Cameroon is in an emerging phase with an increasing demand for digital health technologies, especially in the area of reproductive, maternal, newborn, child, and adolescent health. This article describes and documents the steps in operationalization of the ANC DAK content into the BornFyne-PNMS content, highlighting the DAK as an important tool for guiding and facilitating software engineers in developing and integrating recommended ANC guidelines into digital platforms to facilitate interoperability, going by the structure of the document, its workflow processes, and content mapping elements.

## 1 Background

The adoption of digital health technology is increasingly becoming fundamental in healthcare delivery across several Sub-Saharan African nations. Governments and national organizations have shown considerable enthusiasm toward utilizing digital health and its applications as scalable tools to offer efficient, safe, personalized care to service users (World Health Organization, 2017; Holst et al., 2020; Manyazewal et al., 2021; Murray et al., 2016; Global System for Mobile Communications Association, 2021). Digital health devices and applications have proven instrumental in overcoming geographic, infrastructural, and human resource challenges, while also enhancing health provider performance and patient education (World Health Organization, 2017; Holst et al., 2020; Manyazewal et al., 2021; Murray et al., 2016; Global System for Mobile Communications Association, 2021). These interventions have demonstrated effectiveness in reaching inaccessible locations with limited infrastructure and in enhancing communication across various tiers of the healthcare system (Manyazewal et al., 2021; Murray et al., 2016; Global System for Mobile Communications Association, 2021). Such advancements are particularly significant as they address the challenge of reaching women in underserved rural and remote areas, a common hurdle faced by global health initiatives targeting maternal health services (Global System for Mobile Communications Association, 2021).

The 2018 World Health Assembly Resolution on Digital Health underscored the significance of digital technologies and their role in promoting universal health coverage (World Health Organization, 2018). This resolution urges ministries of health to assess the utilization of digital technologies, such as health information systems, both nationally and sub-nationally, and pinpoint areas needing improvement and establish priorities for the development, evaluation, utilization, scale-up, and expansion of these technologies (World Health Organization, 2018).

In 2020, Cameroon introduced its ambitious digital health framework, acknowledging the crucial role played by digital tools and technologies in addressing and alleviating various challenges encountered by the healthcare system, such as geographic inaccessibility, low service demand, delays in care delivery, poor adherence to clinical protocols, and high out-of-pocket costs (MINSANTE). The framework envisions 70% of health facilities adopting digital solutions by 2024 (pending updates) (MINSANTE). Objectives include enhancing healthcare providers’ capacity to use digital platforms, integrating applications into existing systems, establishing interoperability with DHIS2, and advancing 4G technology (MINSANTE). This framework serves as a guide for stakeholders, funders, and researchers to support Cameroon’s digital health goals.

The World Health Organization (WHO) introduced Digital Adaptation Kits (DAKs) for antenatal care (ANC) in 2021 as part of its Standards-Based, Machine-Readable, Adaptive, Requirements-Based, and Testable (SMART) guidelines initiative (World Health Organization Digital adaptation, 2021; Tamrat et al., 2022; Muliokela et al., 2022; Haddad et al., 2020; Mehl et al., 2021). The DAKs include data dictionaries and decision support tools, providing software-neutral mechanisms to standardize WHO guidelines for easy integration into digital systems worldwide (World Health Organization Digital adaptation, 2021; Tamrat et al., 2022; Muliokela et al., 2022). DAKs offer business process workflows, core data elements, algorithms, and functional requirements to guide the design of point-of-care digital systems (World Health Organization Digital adaptation, 2021). By creating these operational tools derived from WHO guidelines, the DAKs offer a distinctive approach to reinforcing recommendations and establishing linkages in service delivery (World Health Organization Digital adaptation, 2021; Tamrat et al., 2022; Muliokela et al., 2022). By translating WHO guidelines into operational components, DAKs enable countries to accelerate digitalization efforts, improve system design, and streamline the adoption of WHO clinical, public health, and data utilization guidelines (World Health Organization Digital adaptation, 2021; Tamrat et al., 2022; Muliokela et al., 2022).

Maternal morbidity and mortality remain a major public health problem in Cameroon (MINSANTE, 2024; Ministere de la Sante Publique, 2016). Reported maternal mortality rates of up to 596 deaths/100,000 live births are among the highest in the world (World Health Organization, 2019a), attributed to delays in seeking and receiving care, suboptimal quality of care, inadequate adherence to guidelines, and the absence of reliable, actionable data for prompt intervention and decision-making (MINSANTE, 2024; Ministere de la Sante Publique, 2016; Thaddeus and Maine, 1994). To address some of these challenges and to support Cameroon’s vision for digital health, the BornFyne-Prenatal Management System (PNMS)—a two-way interactive digital platform with six modules developed in 2018—was updated to align with the ANC DAK requirements (Nkangu et al., 2020; Obegu et al., 2023). One component is a user-facing smartphone application (referred to as U1), which offers personalized antenatal and postnatal reminders, family planning, and connections to emergency transport services (Nkangu et al., 2020; Obegu et al., 2023; Nkangu et al., 2024a). The other component is a health worker-facing electronic medical record (EMR) (designated as U2), structured to facilitate data entry during antenatal and postnatal visits, ensuring more comprehensive, accurate, and timely data collection (Nkangu et al., 2020; Obegu et al., 2023; Nkangu et al., 2024a). The EMR has been re-designed by integrating the country adapted DAK content for antenatal care in Cameroon. This update seeks to improve the existing BornFyne-PNMS content and support health workers in adhering to guidelines and making informed clinical decisions. Thus, the BornFyne-PNMS platform provides Cameroon with an opportunity to leverage the SMART Guidelines DAK for ANC to advance its digital health vision for maternal health and achieve the strategic objectives outlined in its health framework.

Adopting standardized terminology is crucial for clarity and consistency in digital health platforms (Pretty et al., 2023). Given the limited research and guidance on DAK implementation, documenting the integration steps in BornFyne-PNMS v2.0 is vital for the digital health ecosystem and countries looking to adopt this approach. BornFyne-PNMS is the first reproductive, maternal, newborn, child, and adolescent health (RMNCAH) digital platform in Cameroon to incorporate the DAK elements to enhance antenatal care delivery. This article outlines the methodology used to update and integrate WHO DAK content into BornFyne v2.0, utilizing the DAK data dictionary and decision support components to refine the antenatal care module (World Health Organization Digital adaptation, 2021). Documentation of the steps and approaches used to develop digital platforms integrating the DAK content is important in informing the digital health ecosystem, the research community, and ministries of public health in countries adopting the DAKs. While the development and features of the BornFyne platform have been detailed elsewhere (Nkangu et al., 2020; Obegu et al., 2023; Nkangu et al., 2024a), this article focuses exclusively on the steps taken to update the ANC content within BornFyne 2.0 using the DAK.

## 2 Methodology

### 2.1 Study setting

Cameroon is a lower–middle-income country, with a population estimate of 26 million in 2020 (The World Bank Population Data, 2024). Women comprise approximately 50% of the population as of 2023 data (The World Bank Population Data, 2024). Health is financed mainly through out-of-pocket expenditures (The World Bank Population Data, 2024). The health system of Cameroon is decentralized, with a pyramidal structure comprising the operational, regional, and central levels (MINSANTE, 2024; Ministere de la Sante Publique, 2016). Health policies are defined at the central level by the Ministry of Public Health. The regional level translates the health policies into operations, while the district level operationalizes the health policies into action (MINSANTE, 2024; Ministere de la Sante Publique, 2016; Nkangu et al., 2022). The country has a national health management information system—the dhis2 that collects data in aggregated form for various health outcomes, including maternal health indicators. The BornFyne project is implemented across four districts in Cameroon, purposefully selected to represent both francophone (Ayos, Akonolinga) and anglophone (Tiko, Bangem) regions. Given Cameroon’s heterogeneous health system—a mix of public, private, and confessional health facilities—and its blend of French and English cultures, the pilot includes a diverse range of health facilities to ensure comprehensive integration of DAK content for ANC.

### 2.2 Study design

This study utilized a participatory action research approach, forming part of the larger BornFyne-PNMS project (Obegu et al., 2023; Nkangu et al., 2024a). This approach, ideal for co-developing research with stakeholders rather than for them (McIntyre, 2008), was crucial for integrating DAK content into BornFyne. Given the challenges of transitioning from paper-based systems to digital platforms, involving health providers, administrators, and software engineers from planning to testing was critical to ensure relevance and contextual factors were taken into account. As some of the generic WHO DAK elements may not align with specific contexts and the entrenched practice of using paper, limited resources, and other possible challenges (Tamrat et al., 2022; Haddad et al., 2020; Kawamoto et al., 2005; Puchalski Ritchie et al., 2016), we considered these important contextual differences that can arise when applying global guidelines. This is relevant where the contextual realities of the community in question may be quite distinct from those of the people designing and formulating the policies and guidelines that will affect people’s lives and daily practices. Workshops, demo testing, and consultations gathered stakeholders’ feedback on using guidelines and decision support tools. Additionally, engagement with WHO teams provided insights for updating BornFyne-PNMS v2.0 to reflect local realities and needs.

### 2.3 Overview of the steps in updating the BornFyne content

**FIGURE 1:**
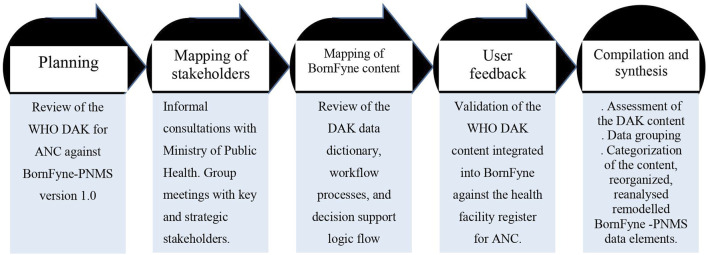
Overview of the five phases followed to update the BornFyne-PNMS content using the DAK for ANC guidelines.

The content integration process employed a phased approach, which meant breaking down the activities into smaller tasks to facilitate implementation (Banas et al., 2011). This was divided into five steps: planning, mapping of stakeholders and consultations, mapping of content and consultations, user feedback and validation, and content compilation and synthesis (see Figure 1 below).

#### 2.3.1 Planning

The planning phase involved reviewing the WHO DAK for ANC by the BornFyne research and development teams to identify and compare the DAK data dictionary and decision support tool elements with the existing content in BornFyne-PNMS v1.0 and any normative guidelines for ANC (if available) and/or health facility registers. This step was crucial because (1) BornFyne-PNMS v1.0 was developed prior to the launch of the DAK for ANC, (2) the BornFyne platform initially used the WHO focused antenatal care package as a guide in developing the ANC module, necessitating a comparison of its elements with the DAK and health facility registers, (3) potential differences between health facility registers and DAK guidelines, and (4) resource constraints by the BornFyne team that limited the ability to fully integrate the DAK content. Thus, the team prioritized calling existing BornFyne data elements with those in the DAK. The content was mapped with software engineers to define the project scope, considering resource limitations and the implementation timeframe.

#### 2.3.2 Mapping of stakeholders

We identified stakeholders essential for implementing the digital health intervention (World Health Organization, 2019b). These included healthcare providers delivering maternal health services (nurses, doctors, and midwives), health facility administrators, district medical officers, the regional delegation team, and central-level representatives from the Ministry of Public Health, specifically the Department of Family Health. At the central level, stakeholders were identified by the Department of Family Health at the Ministry of Public Health. While outcomes of stakeholder meetings for the broader BornFyne-PNMS project are detailed elsewhere (Obegu et al., 2023; Nkangu et al., 2024a; Nkangu et al., 2024b), this article focuses solely on documenting the steps for integrating the DAK content for ANC into BornFyne-PNMS and scoped the outcome of the stakeholder discussions that were relevant to the WHO DAK components for the BornFyne ANC module.

Workshops with health providers were conducted to align BornFyne-PNMS content with DAK guidelines and address differences, including those in existing health facility registers. Mapping was conducted exclusively with health providers at the health facilities using the health facility registers, given the absence of normative ANC guidelines at the health facilities. This process was followed by consultations with other stakeholders. The stakeholder meetings involved both informal consultations and group meetings with relevant and strategic stakeholders. “Relevant stakeholders” in this study are those identified by the Ministry of Public Health, for example, district medical officers, health providers, data units, and selected directors from different departments within the Ministry as necessary for implementing digital health projects in the context of RMNCAH. “Strategic stakeholders” are a subset of the relevant stakeholders identified by the project team during the central-level stakeholder meetings to engage in follow-up and one-on-one consultations on content-specific items. For example, the data unit responsible for DHIS2 offered valuable insights into the contextual and national indicators, as well as existing variables, while others contributed their expertise to previously integrated guidelines developed for RMNCAH. The strategic stakeholders were identified with the assistance of the designated focal point for the BornFyne project within the Directorate of Family Health.

#### 2.3.3 Mapping BornFyne-PNMS ANC content against DAK content

A series of meetings were held with the BornFyne-PNMS research and IT teams to map the data elements of the existing BornFyne-PNMS v1.0 features. Most of the meetings with the IT team were conducted remotely. The IT team held weekly meetings, while the research team convened bi-weekly. Additionally, the research and IT teams met twice a month for collaborative discussions. The team conducted a review of the data dictionaries, workflow processes, and decision support logic to assess alignment with existing components and identify areas of divergence. A consensus was reached, and a list of elements was generated. This step was essential to ensure alignment between the software engineers and the research team before engaging stakeholders, ensuring a clear and organized scope of work.

Subsequently, a stakeholder meeting was convened to introduce the DAK, discuss its features and elements, and identify strategic stakeholders for further involvement. This meeting served as an orientation to the DAK, focusing on familiarizing participants with its content. Stakeholder meetings were conducted at multiple levels, followed by continuous consultations to present the DAK data elements and the BornFyne v1.0 and gather insights into our proposed approach to updating and expanding the existing components to align with the recommended core data elements within the scope and timeframe of the project. The focus was placed on updating existing components using the recommended data elements and resolving discrepancies between BornFyne content and the DAK standards that integrate International Classification of Diseases (ICD) codes. Discussions with stakeholders helped address these differences, ensuring alignment with global standards while recognizing that many existing variables in the health facility registers lacked detailed specifications but conformed to ICD standard. Considering that the existing variables in the health facility registers collected during ANC lack detailed specifications, most of these elements align with the guidelines outlined in ICD. To gather comprehensive feedback, we organized stakeholder meetings, one-on-one consultations with strategic stakeholders, workshops, and group discussions.

Subsequent consultations identified and mapped out the existing variables, definitions, and indicators collected at the health facility, the system for ANC and the standard procedures for ANC at health facilities in the target districts. This step focused on generating an understanding of the elements of the DAK and identifying the components currently in the BornFyne-PNMS, the gaps or areas that needed updating, and how to go about updating the identified elements. This step focused on aligning existing BornFyne content systematically with their ICD codes, including incorporating additional questions taken from the DAK content to provide clarity to the existing BornFyne content and to develop additional layers that describe the activity name and data element ID. It also defined the additional features to be incorporated, given the scope of the project and limited funding.

#### 2.3.4 User feedback and validation of the DAK content integrated into BornFyne against the health facility register for ANC

Phase IV involved selected health providers who provide routine antenatal care. These health providers were asked to review the DAK against the health facility registers and provide feedback. The feedback aimed to validate the items integrated into the BornFyne-PNMS against the health facility register. The research and IT teams collaborated to map the BornFyne content against the DAK, and health providers validated this mapping. Health providers mapped the DAK content against the health facility register when the research team facilitated the exercise during a workshop. This process was critical considering that the planning and mapping phase identified an important gap that indicates the absence of normative ANC guidelines at health facilities; thus, the reference document used was the health facility registers. This important observation on the absence of ANC guidelines at health facilities was further discussed and explored during the workshop that assessed the DAK content against the existing health facility register. The outcome of the workshop on the DAK content (against the health facility register for ANC) and the integration into BornFyne and possible pain areas are analyzed and presented as a separate article, which will be a sequel to this article (Nkangu et al., 2024). A list of items was identified, documented, and shared with the WHO team for feedback and directions. Health providers provided feedback to the software engineers, taking into consideration the contextual challenges to facilitate data entry. A comparison of the elements that are entered into the daily register for antenatal care was made against the existing elements within the BornFyne-PNMS to assess the differences and amount of data to be included in their workload as the team pilot test of the BornFyne-PNMS.

#### 2.3.5 Data compilation and synthesis

We assessed the content of the DAK and grouped the data from the BornFyne-PNMS version 1.0 according to the DAK framework. We further categorized the content of the discussions and consultations with stakeholders. We used the DAK data elements to group the elements in version 1.0 into each category using the DAK categorization alongside the assigned ICD codes (Supplementary Figures S2, S3) and then generated outputs that were shared with the WHO team and stakeholders for feedback. We listed the existing elements in BornFyne-PNMS version 1.0 and grouped the elements according to each category as defined in the data dictionary. Then, we identified and agreed on the elements to be updated, developed, and expanded upon within the timeframe of the project. The data elements were reorganized, reanalyzed, and remodeled (see Supplementary Table S1). Finally, we harmonized the content from the stakeholder meetings and coded the content to identify stakeholder perspectives of the DAK tool, potential challenges, and facilitators in the implementation. We harmonized the outcomes of the stakeholder meetings and the review process of the DAK tool (see Table 3).

### 2.4 Ethics approval

Ethical approval was obtained from the National Ethics Board of Cameroon ref # 2022/07/1467/CE/CNERSH/SP and the University of Ottawa Social Science Ethics Board ref #H-05-22-8077. Administrative clearances were obtained from the Ministry of Public Health at the national level in Cameroon (ref. D30-1440 No. 631-3822) in collaboration with the Division for Health Operations Research (DROS) in Cameroon, the Southwest Regional Delegation of Public Health (ref. P412/MINSANTE/SWR/RDPH/CB:PF/941/618), and the Central Regional Delegation of Public Health (ref. 1393-4/AAR/MINSANTE/SG/DRSPC).

## 3 Results

There were five stakeholder meetings, including two at the central level. A total of 119 stakeholders were engaged in the process, including the Department of Family Health at the Ministry of Public Health, the district and regional levels, healthcare providers, software engineers, and the research team, as listed in Table 1 below. Further engagement with the WHO country office to inform the ongoing development process led to the presentation of the BornFyne-PNMS project and to the introduction of the DAK tool at the national coordination meeting for RMNCAH in Cameroon.

**TABLE 1:** Characteristics of stakeholders engaged in integrating the DAK content into BornFyne-PNMS version 2.0.

Health system structure	Stakeholders	Number of stakeholders engaged in the process of phase I
District level (Akonolinga, Ayos, Bangem, and Tiko)	Health providers & district health teams (medical doctors, district medical officers, nurses, midwives, health facility administrators, dhis2 data managers)	67
Regional level (Tiko, Buea, and Yaounde)	Regional delegation team, dhis2 representative, performance-based financing representatives	12
Central level (Yaounde)	The directorate of the family health team at the Ministry of Public Health, representatives from independent organizations and sectors relevant to RMNCAH identified by the Ministry, and the digital health and data unit	17
IT team	Software engineers (app and software developers), data managers, and graphic designers	5
BornFyne research team	Epidemiologist, medical practitioners, health economist, anthropologist, public health practitioners, global health practitioners, and population health	18

### 3.1 Mapping of stakeholders

#### 3.1.1 Stakeholder meetings and consultations

One central-level stakeholder meeting and four district stakeholder meetings were held. This included prior consultations with the regional delegation of health in the central and southwest regions, the district medical officers for each selected district, and the Directorate of Family Health at the Ministry of Public Health. As BornFyne-PNMS has been implemented in Cameroon since 2018, the approach involved updating the features and expanding them to incorporate the DAK guidelines. A demo version of the variables included in BornFyne-PNMS v1.0 (see Table 2) and the identified DAK elements to be expanded in version 2.0 were presented to the stakeholders at the central level. The scope of the integration items from the DAK data dictionary was revisited for remodeling, input, and feedback. The manuals for version 1.0 were then shared with stakeholders, along with other materials. The aim was to capture the definition of variables, understand the variables’ acceptability, and ensure that they were easy to use.

**TABLE 2:** Content of WHO DAK for ANC in BornFyne-PNMS version 1.0 and identified elements for expansion in v2.0.

WHO DAK components	Summary description of WHO DAK for each component	DAK elements existing within BornFyne-PNMS version 1.0	Identified elements for integration into version 2.0	Brief overview of contextual observations in the planning phase
1. Generic persons	Healthcare providers engaged in care delivery (nurses, doctors, community health workers, etc.)	Already built with this scenario in mind with various administrative layers that allow the creation of user accounts for healthcare providers (doctors, nurses, midwives), facility administrators, district medical officers, and regional or national administrators with specific roles and privileges	BornFyne-PNMS v1.0 was already built with this scenario in mind	Mostly, nurses and midwives who attend to routine antenatal care at the health facility, and in some cases, where there is a need for referral to the doctor and gynecologist. Community health workers (CHWs) support referral programs at the household level for women to attend ANC
2. User scenarios	End-users, supervisors, and related stakeholders who would be interacting with the digital system	Some of the interactions that may take place during ANC.A registration and ANC.B routine contact and some workflows existed.	Built with the scenario in mind with various administrative layers. In addition to interactions by generic person (health providers) with the community (women and household), as a 2-way communication during ANC	Paper system at a health facility. Pregnant women have ANC registration cards, CHWs support referrals for ANC, health providers enter ANC data on facility registers, data managers enter indicators in aggregate format into dhis2 and other systems
3. Generic business processes and workflows	A set of related activities or tasks performed together to achieve the objectives of the health programme area. Workflows are a visual representation of the progression of activities (tasks, decision points, interactions) that are performed within the business process	ANC.A. Registration ANC.B. Routine ANC Contact -Record health history -Assess danger signs Workflow was not broken down into processes	Need to remodel existing content and processes in BornFyne to include additional data elements for case management or referral, schedule follow-up, physical exam, and ANC.C. Referral	Paper system at the health facility, but not all elements listed in the DAK are observed in the facility register In addition, the need for additional discussion with stakeholders for consensus to validate and contextualize. The team requires additional resources to support this process
4. Core data elements	Data elements are mapped to the International Classification of Diseases version 11 (ICD-11) codes and other established concept mapping standards	Some elements existed for ANC.A Registration - ANC.B5 Quick Check - ANC.B6 Profile - ANC.B8 Physical Exam but none was defined with an activity ID or ICD code	Remodel and expand on all data elements from - ANC.A Registration - ANC.B5 Quick Check - ANC.B6 Profile - ANC.B8 Physical exam and map out ICD codes	Paper system at the health facility, but not all elements listed in the DAK are observed in the facility register No ANC normative guidelines at health facilities. An introductory one-page (first page of the register) to guide how to complete the register with some definitions
5. Decision support logic	Decision support logic and algorithms to support appropriate service delivery in accordance with WHO clinical, public health, and data use guidelines	Selected elements existed using a decision tree for risk stratification to flag high-risk pregnancies, for example, history of preeclampsia, age, adherence to malaria pills, and blood pressure. Need additional resources to update this feature	Need to remodel and expand on the decision support system and risk stratification for high-risk pregnancies. Require additional resources to support this process	Paper system at the health facility, but not all elements listed in the DAK are observed in the facility register Requires further discussions with district and central-level stakeholders for consensus. Additional resources needed by the team to support this process
6. Indicators and performance metrics	Core set of indicators that need to be aggregated for decision-making, performance metrics, and subnational and national reporting based on data that can feasibly be captured from a routine digital system	Data are in disaggregated format. Some but not all indicators exist; need additional resources to support this process	Require additional follow-up meetings with strategic stakeholders for consensus. Additional resources needed to complete this process	Paper system at the health facility, but not all elements listed in the DAK are observed in the facility register. Aggregated data for specific indicators summed up from paper registers are entered into dhis2 Requires further discussions with district and central-level stakeholders for consensus, and the team needs additional resources to support this process
7. Functional and non-functional requirements	List of core functions and capabilities the system must have to meet the end-users’ needs and achieve tasks within the business process. Non-functional requirements provide the general attributes and features of the digital system to ensure usability and overcome technical and physical constraints	Some elements existed but not systematically defined	Require additional follow-up meetings with strategic stakeholders for consensus on some items. Additional resources needed to complete this process	The team needs additional resources to support this process

The first two columns are from the DAK tool^8^.

To ensure that the stakeholders remained committed throughout the development and implementation of the project, the stakeholders at the central level proposed the creation of a WhatsApp group to coordinate communications, share updates on the project, and disseminate quick queries at the central level. At the district and health facility, another WhatsApp group was created for health providers engaged in the training and implementation and for the software engineers and project team. Table 3 illustrates the outcomes of the stakeholder meetings and consultations in the integration process in detail. Additionally, a soft copy of the WHO DAK data dictionary was shared with the Directorate of Family Health and the central stakeholders in the WhatsApp group. After the stakeholder meetings, we identified strategic stakeholders with whom to follow up regarding the data elements and discussed with the digital health unit and DHIS2 representatives. In this process, we engaged other strategic stakeholders responsible for data and data management, the RMNCAH representatives at the Ministry of Public Health, in an ongoing discussion regarding indicators, performance metrics, and interoperability. We continue to exchange feedback with the team at WHO on our experiences with implementing the DAK for ANC in the BornFyne-PNMS v2.0 in the context of Cameroon.

**TABLE 3:** Outcome of stakeholder meetings and the five steps (PMUCs) involved in integrating the WHO DAK components for antenatal care within the BornFyne-PNMS platform.

	Methods/Approach	Nature of activity and/or document reviewed	Description	Purpose	Output/Outcome	Adaptation elements	Remarks or observations from the software team and/or stakeholders
Step I: Planning							
Review	Review of WHO DAK documents and consultations with medical experts and engaged WHO team	DAK data dictionary, decision support logic, indicator document.	Identify and list elements within BornFyne-PNMS that are within DAK	To generate a document to guide the software team on elements for integration	Generated a document with DAK elements to be updated and expanded within the scope of the project and timeframe	Focused on content and context	DAK data dictionary, decision support, and indicator tables are in English and required translation into French
Step II: Mapping of stakeholders and consultations							
Stakeholder mapping	Engaged stakeholders at regional, central, and district levels Informal discussions with WHO at the international and country level	Formal and informal meetings with key stakeholders at central, regional, and district levels discussions on DAK components	Stakeholders from the Ministry of Public Health at the central, regional, and district levels	Presentation of BornFyne-PNMS as a digital health tool and introducing the WHO DAK elements for ANC to the central level stakeholder team aligning with the national digital health framework and the objective of addressing maternal health issues within the broader context of achieving universal health coverage and strengthening health management information system	WhatsApp forum of key stakeholders at the central level was created to collect feedback and monitor the progress of the project Established a defined and coordinated partnership between the BornFyne-PNMS project and the Department of Family Health at the Ministry of Public Health for the pilot implementation in the four districts	Focused on contextual challenges	High interest in data alignment with existing dhis2 and clear definition of variables in line with existing facility registers. No ANC normative guidelines at health facilities. Elements in the health facility registers conform with ICD guidelines but are not detailed and are not coded
Step III: Mapping of content and consultations							
Meetings with research and IT team	Continuous process. Follow-up meetings with strategic stakeholders identified during the stakeholder meetings	One-on-one meetings with relevant stakeholders as needed to inform the intervention and development process	Share soft copy of DAK data dictionaries with stakeholders for review and feedback	Focus on data elements as listed in the DAK data dictionary adapting to context and the existing facility registers for antenatal care at health facilities	Project team agreed on the list of existing elements to be updated and defined within the scope of the project and timeframe	Focused on elements in the health facility register and any differences	Additional consultations and workshops to assess the absence of ANC guidelines at health facilities and to assess facility registers against the DAK
Update and expand the BornFyne-PNMS version 1.0	Continuous process. Follow-up meetings with the software team to discuss feedback and identify intervention approaches for remodeling	List data elements from the DAK data dictionary to be analyzed	Remodel, reorganize, and reanalyze existing elements to comply with DAK recommended data element ID and ICD codes (see Table 4)	To align with recommended guidelines in the DAK data dictionaries and identify possible contextual differences	Generate outputs for review and feedback (see Figures 2, 3)	Focused on potential contextual differences in care delivery	Time and volume of elements to be analyzed within the timeframe
General testing	Testing of data elements	Testing with the research team and health providers	Testing on preproduction site and feedback	An iterative process to provide feedback on platform usability and ease, especially the translated components	Generated outputs	Identified contextual differences and challenges	Identify pain areas when using the digital platform to deliver care and adherence to guidelines
Demo testing	Demo testing	Testing with the research and IT teams using preproduction sites	3–4 h testing using BornFyne-PNMS v2.0 for antenatal care by the project team	Define training manuals focused on those engaged in daily care delivery of antenatal care and guidelines, including health facility administrators and data managers	Develop training manuals for review	Focused on practical day-to-day contextual challenges at health facilities	List of items for adjustment to context. Relevant to support training and build capacity
Consultations with strategic stakeholders	Follow up with strategic stakeholders identified during the stakeholder meetings	One-on-one meetings with relevant stakeholders based on the need to inform the intervention and integration process	Stakeholders related to specific project items, data, dhis2, definition of indicators, and developing messages.	Focus on data elements as listed in the DAK tool and any existing country normative guidelines and indicators for antenatal care at health facilities	Identify existing working documents on variables for performance metrics and interoperability standards	Focused on context at the system delivery level	Possible challenges in using the existing DAK elements within BornFyne. Pain areas and recommendations at the system level for consideration
Step IV: User feedback							
Consultations	Consultations and meetings with health providers	Health providers engaged in RMNCAH at health facilities, using the BornFyne-PNMS platform	Discussed workflow processes and potential challenges based on context	To validate workflow processes and data elements versus facility register	Identify elements and differences in health facility registers and integrated DAK elements in BornFyne	Focused on contextual challenges	Document feedback and recommendations for the BornFyne-PNMS workflow and next steps
Organizing workshop^a^	Workshop with health providers on using BornFyne-PNMS	Selected health providers engaged in antenatal care delivery	4–5 h per district on the integrated DAK elements	Workshop to validate workflow processes and data elements, including facility register	Generate feedback to support development components and workflow processes	Focused on contextual challenges at health facilities	Challenges in using the existing DAK elements within BornFyne. Pain areas and recommendations at the facility level
Step V: Compilation and synthesis							
Update and expand the BornFyne-PNMS version 1.0	Continuous process. Follow-up meetings with the software team to discuss feedback and identify intervention approaches for remodeling	List data elements from the DAK data dictionary to be analyzed	Remodel, reorganize, and reanalyze existing elements to comply with DAK recommended data element ID and ICD codes (Table 4)	To align with recommended guidelines in the DAK data dictionaries and identify possible contextual differences	Generate outputs for review and feedback {see Figures 6 & 7)	Focused on potential contextual differences in care delivery	Time and volume of elements to be analyzed within the timeframe
	Feedback incorporated and compiled	Supported by peer-to-peer testing	Observe and support health provider’s peer-to-peer facilitation and presentation to stakeholders	Focused on adherence challenges, timing, facilitators, and workflow vis-a-vis existing normative guidelines or facility register for antenatal care at health facilities	Identify and document differential variables between DAK and facility registers and BornFyne	Discussed differential variables and explored opportunities and dimensions	Document feedback and recommendations for the BornFyne-PNMS project and next steps

Continuous and iterative processes and feedback.

^a^ Workshops and outcomes of the workshop are described in detail in a separate article.

**TABLE 4:** Selected data content from the DAK included in the BornFyne-PNMS v1.0 versus BornFyne-PNMS v2.0, including the ICD codes.

ANC registration						
[ANC] activity ID	[ANC] data element ID	ICD-11 Code	Data element label	Description and definition	BornFyne version 1.0	BornFyne version 2.0
ANC.A7. Create client record OR ANC.A8. Validate client details	ANC.A7.DE1	Not classifiable in ICD-11	Unique identification	Unique identifier generated for new clients or a universal ID, if used in the country	P	P
ANC.A7. Create client record OR ANC.A8. Validate client details	ANC.A7.DE2	Not classifiable in ICD-11	First name	Client’s first name	P	P
ANC.A7. Create client record OR ANC.A8. Validate client details	ANC.A7.DE3	Not classifiable in ICD-11	Last name	Client’s family name or last name	P	P
ANC.A7. Create client record OR ANC.A8. Validate client details	ANC.A7.DE4	Not classifiable in ICD-11	Contact date	The date and time of the client’s contact	A	P
Quick Checks						
ANC.B4. Confirm pregnancy	ANC.B4.DE1	XT0S	Pregnancy confirmed	Pregnancy has been confirmed	A	P
ANC.B5. Quick check	ANC.B5.DE1		Reason for coming to the facility	Records the reason why the woman came to the healthcare facility today		
ANC.B5. Quick check	ANC.B5.DE2	Not classifiable in ICD-11	First antenatal care contact	This is the woman’s first ANC contact	A	P
ANC.B5. Quick check	ANC.B5.DE3	Not classifiable in ICD-11	Scheduled antenatal care contact	The woman is coming in for a scheduled ANC contact	A	P
ANC.B6 Profile						
ANC.B6. Collect woman’s profile and history	ANC.B6.DE1		Highest level of education achieved	The highest level of schooling the woman has reached		
ANC.B6. Collect woman’s profile and history	ANC.B6.DE2	Not classifiable in ICD-11	Does not know level of education	Woman does not know the level of education they have received	P	P
ANC.B6. Collect woman’s profile and history	ANC.B6.DE3	Not classifiable in ICD-11	No education	Woman has received some primary education or no primary education	P	P
ANC. B8 Physical Exams						
ANC.B8. Conduct physical exam			Height and weight			
ANC.B8. Conduct physical exam	ANC.B8.DE1	Not classifiable in ICD-11	Height	The woman’s current height in centimeters	P	P
ANC.B8. Conduct physical exam	ANC.B8.DE2	Not classifiable in ICD-11	Pre-gestational weight	The woman’s pre-gestational weight in kilograms	A	P
ANC.B8. Conduct physical exam	ANC.B8.DE3	Not classifiable in ICD-11	Current weight	The woman’s current weight in kilograms	P	P
ANC.B8. Conduct physical exam	ANC.B8.DE4	Not classifiable in ICD-11	BMI	Body mass index (BMI): calculated by taking weight in kg divided by the squared height in meters; that is, kg/(m^2^)	A	P

The first five columns are from the DAK. A = Absent: P=Present (no data element of ICD, code in BornFyne-PNMS, version 1.0, updated version 2.0 has data element ID and ICD codes). Note this table does not capture all the elements that have been integrated into BornFyne-PNMS, v2.0. See Supplementary Table S1 for additional information.

#### 3.1.2 Outcome in mapping the content of the WHO DAK for ANC into BornFyne-PNMS v2.0

##### 3.1.2.1 ANC DAK operational guidelines and data dictionary

**FIGURE 2:**
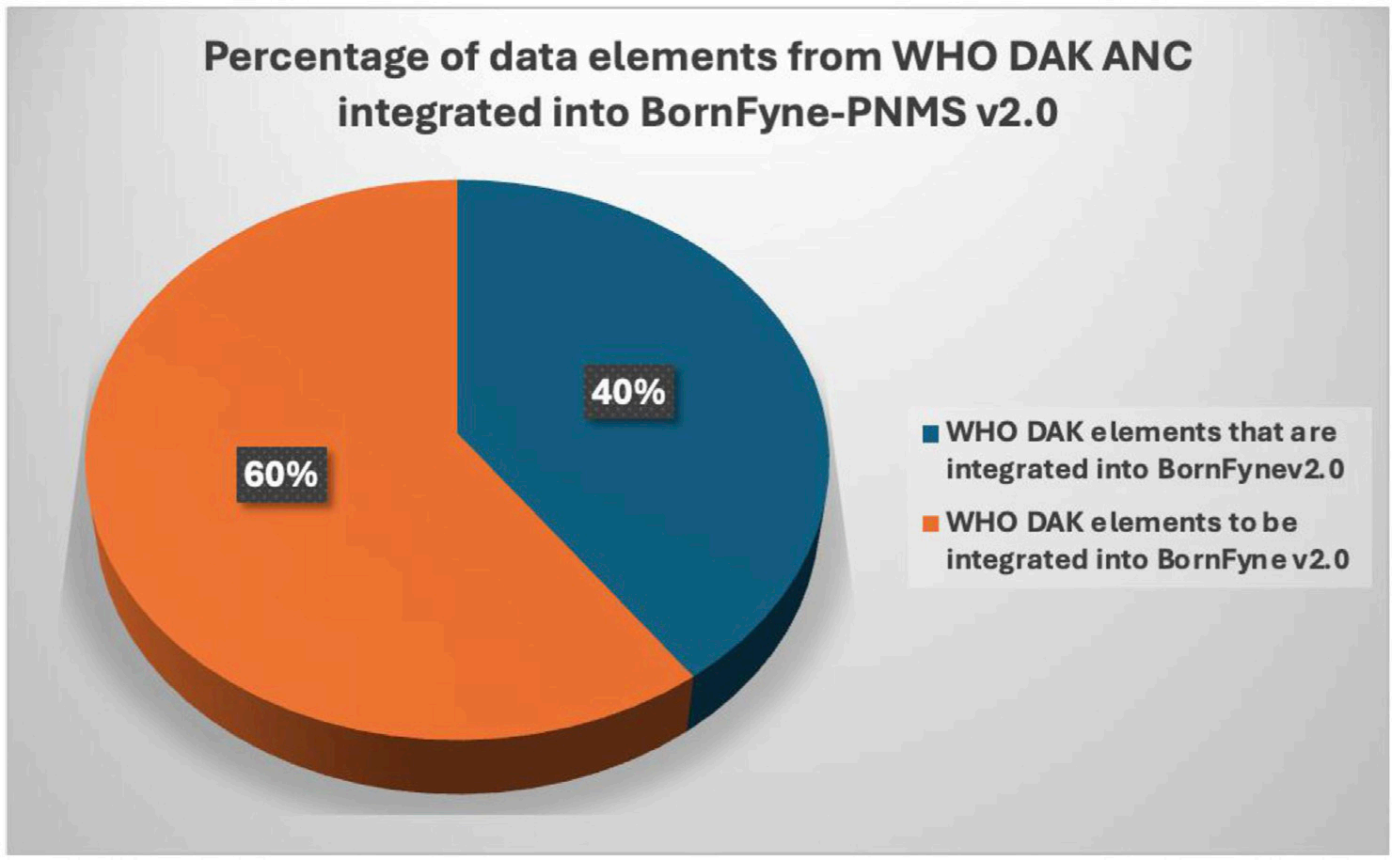
Percentage of data elements from the WHO DAK for ANC integrated into the BornFyne-PNMS.

It was observed by the software engineer’s assessment that approximately 40% of the functional and non-functional requirements specified for the antenatal care package were already incorporated into BornFyne-PNMS v1.0 (Figure 2). The team reviewed the DAK operational guidelines and data dictionaries and listed the elements among the 40% (including non-functional requirements) of the data elements that existed within the BornFyne-PNMS v1.0. These included the registration process, profile and history, physical exams, the two-way interactive mode, the community aspect, and the decision tree system already established to flag high-risk pregnancies (limited to those of HIV-positive women, malaria-related variables, and women with other pre-existing conditions). It should be noted that the existing risk stratification decision tree system in BornFyne version 1.0 was identified as a component to be updated using the DAK-recommended approach. The team could not update this component in this pilot phase due to limited resources but noted it for subsequent updates. In addition, the existing BornFyne risk stratification content was not initially coded using the recommended data element IDs and ICD codes. Thus, the team listed the features to be updated and included additional data elements to expand on the additional features within the defined scope. Table 2 provides an overview of the high-level elements that are listed within the DAK data elements and decision support and provides an overview of what was observed to be present in the BornFyne v1.0 that needed to be reorganized, remodeled, and reanalyzed in addition to elements that were identified by the team to be added into the version 2.0 as the software team proceeded with reorganizing the elements.

### 3.2 Result of the feedback phase

Selected health providers further tested the platform, and an output was shared with the WHO team that illustrates how the development and the ICD codes are configured for the existing data elements, as shown in Supplementary Figures S3, S4. The first output from the software engineers (Supplementary Figures S1, S2) was reviewed by the team, and some items were marked for discussion during the workshop with health providers to capture contextual differences and practical challenges, taking into consideration the French and English approaches in delivering antenatal care within each district. The detailed outcome of the workshop is described separately.

**FIGURE 3:**
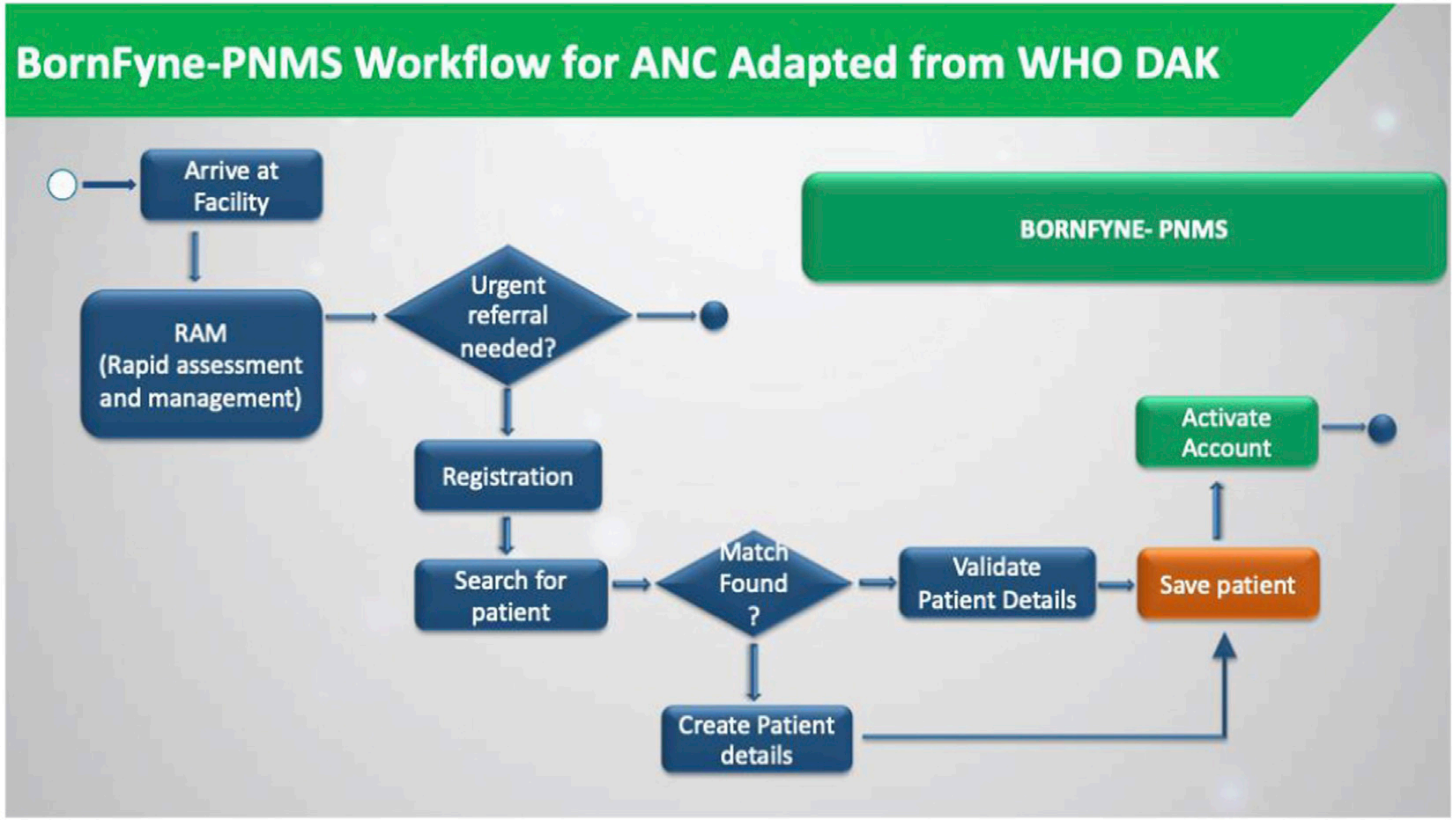
The BornFyne-PNMS workflow was adapted from the DAK decision support workflow. The workflow is only for the registration module.

The team revised the existing workflow in BornFyne, which was initially not divided into distinct workflow processes. For example, registration and patient profiles were all collected in a single workflow. There was no quick check in BornFyne version 1.0. The DAK decision support logic guided the process of breaking these down into separate workflows. Consequently, BornFyne version 2.0 adopted DAK’s structured approach, as illustrated in Figure 3. However, it is important to note that providers emphasized the need for simpler workflows and provided feedback to ensure the system could be easily adapted to their specific context and available resources. One key difference in the BornFyne workflow, highlighted in green, is the ability for the provider to activate the client immediately after registration. This activation links U1 to U2, effectively connecting the woman to the health facility and granting her access to all six features of the U1 interface.

### 3.3 Results from compilation and synthesis: reorganizing, reanalyzing, and remodeling BornFyne-PNMS data elements

The core ANC data elements in BornFyne-PNMS version 1.0 lacked systematic mapping and integration based on the ICD. The DAK guidelines provided a framework for the BornFyne digital health team to ensure consistency and accuracy in updating health content within the ANC module. The compilation phase involved reorganizing, reanalyzing, and remodeling the existing BornFyne-PNMS v1.0 elements to align with the DAK, referred to as v2.0. This action phase was an iterative process that involved the healthcare providers, IT, and the research team, including other stakeholders and the WHO team through consultations and meetings. In this phase, the identified data elements were organized according to the DAK content. This was organized and developed by the software engineers. The research team tested the first output on the preproduction website. This was reviewed to ensure the items aligned with the elements as stated in the data dictionaries.

Looking at the DAK generic business processes and core data elements listed in Table 3, most of the features for registration already existed in the BornFyne platform, although no activity ID was assigned. The DAK tool helped reorganize the flow of these elements. For example, the name of the client, age, and date of birth were collected in BornFyne v1.0 but lacked an activity label for each data element. With the integration of the DAK data content into the BornFyne content, the first name of a patient is now labeled as activity ANC.A4.DE2 and last name is ANC.A4.DE3 with corresponding ICD codes (Tamrat et al., 2022). This is essential and will facilitate interoperability. In addition, the BornFyne-PNMS version 1.0 generated unique identifiers for each patient but did not assign an activity label. Using the DAK tool, it is currently labeled as ANC.A4.DE1, and date of birth is labeled ANC.A4.DE5, etc.

The team agreed to work on some of the updated elements based on the availability of resources: registration, quick checks, patient profiles, and physical exams. For example, elements such as fever, pain, vomiting, etc., listed in the DAK as fever (ANC.B5.DE53), severe headache (ANC.B5.DE57), severe vomiting (ANC.B5.DE59), severe pain (ANC.B5.DE58), etc., under quick checks, and anti-retroviral ANC.B6.DE119) or anti-malarial (ANC.B6.DE118) under the profile were collected in BornFyne v1.0 as part of physical exams or medication history. These were categorized in BornFyne v1.0 for providers to document in a single cell. However, the DAK has broken these elements down further and assigned data element IDs and corresponding ICD codes, which facilitated the work of both the research and development teams (see Supplementary Table S1). Supplementary Table S1 provides an overview of data elements that have been remodeled to align with the DAK, and the outputs in Supplementary Figures S3, S4 illustrate how the elements are presented within the back end of the BornFyne-PNMS platform.

### 3.4 Quick checks

#### 3.4.1 Client details

##### 3.4.1.1 Differential elements identified in the integration process between health facility registers, BornFyne, and DAK

The antenatal care feature of BornFyne v1.0 was developed by adapting the World Health Organization’s focused antenatal care package (World Health Organization, 2002). The antenatal care feature of BornFyne-PNMS (v1.0) uses a decision tree system to stratify high-risk pregnancies using risk-factor linkage (history of pre-eclampsia, age, and hypertension, among others) to automatically flag high-risk pregnancies, thus facilitating early detection and timely referrals. Given that the BornFyne platform collects details about the use of mosquito nets by pregnant women, adherence to scheduled intermittent presumptive treatment of malaria (IPTM), and adherence of HIV-positive pregnant women to antiretroviral therapy, both primary care physicians and public health experts can be armed with data to inform policy and guide interventions. The variables related to malaria in pregnancy within the DAK focused on prevention counseling and provision of IPTM, and therefore the team could not remodel elements within Bornfyne for other variables related to malaria in pregnancy, such as testing and diagnosis, as it required an additional workshop to validate the questions with stakeholders, define the data element IDs, and identify ICD codes. This observation was also discussed with the WHO team for additional feedback.

### 3.5 Challenges in the integration process

The software engineers faced several key challenges, including domain-specific limitations in understanding advanced clinical and medical terms, as they were not from the medical field. Continuous meetings with the research and medical teams helped clarify these terms, enabling the implementation to proceed. Adapting the existing BornFyne-PNMS v1.0 system to align with DAK guidelines required reorganizing, reanalyzing, and remodeling ANC elements, which was challenging given the project’s timeframe and resources. Although the DAK is available in French and Spanish, its data dictionary has not yet been translated into French. While the research and development teams who are bilingual managed this through consultations to ensure accurate translation, the process underscored the need for professional input in translating technical medical documents.

A major challenge was the time-intensive process of reviewing the DAK content, particularly the decision support logic, ensuring alignment across stakeholders, and obtaining expert clarifications. The team focused on creating a simplified, user-friendly workflow for medical personnel to record patient details in compliance with DAK recommendations, particularly in resource-limited settings where pilot testing occurred. The project’s primary goal of building a decision support system based on recorded information and key indicators required careful integration of DAK recommendations and contextual limitations.

One critical lesson learned was the difficulty of aligning an existing platform like BornFyne with the DAK in the absence of normative ANC guidelines, which significantly increased the team’s workload. Financial constraints further limited the integration of all DAK elements and hindered the team’s ability to support the Ministry of Public Health in contextualizing and adapting a normative ANC guideline using the DAK framework.

### 3.6 Facilitators of the integration process

Some important facilitators observed by the research team during the stakeholder meetings are highlighted. The use of the DAK as a standard guideline for updating the design and implementation of the BornFyne-PNMS digital platform was identified as a facilitator, paving the way for meeting with key and strategic national stakeholders, especially at the central level. This facilitation was mostly due to the fact that the tool was developed by the WHO and partly because we engaged the local WHO office from the onset. Furthermore, the research team observed that the use of the WHO DAK itself was a facilitator in engaging stakeholders who were interested in improving standards and guidelines in order to improve the quality of RMNCAH care. Second, it is important to introduce innovations and digital platforms such as BornFyne-PNMS that target important contextual issues in relation to maternal mortality, which has been a health system burden for many decades, as this allows stakeholders to participate in resolving issues that affect the health system at large. Lastly, we observed that standardization of data and streamlining the inputs for the health management information system helps to improve the reliability and quality of data generated for health systems attempting to strengthen their health management information systems. This was observed mostly because of the absence of normative ANC guidelines to ensure a systematic approach in data collection; therefore, health facilities or health providers collect data in various registers and patient booklets and then aggregate the data into dhis2. This is prone to errors, is labor-intensive, and does not capture and provide reliable data (Nkangu et al., 2024c). Therefore, introducing innovations that align with the country’s priorities and have the potential to affect the entire community provides an avenue for engaging and collaborating with stakeholders to facilitate implementation.

BornFyne-PNMS was strongly accepted at all levels of the health system as a digital health tool that integrates the WHO DAK elements for ANC. This was because of BornFyne’s alignment with the national digital health framework, and the DAKs for ANC clinical content align with the content in the standard paper registers (which conforms with ICD) used at the health facility, all geared towards the objective of addressing maternal health issues within the broader context of achieving universal health coverage and strengthening the health management information system.

## 4 Discussion

This study involved a review of the WHO DAK, followed by stakeholder meetings and consultations focused on the steps in the integration of the DAK content within the existing BornFyne-PNMS ANC module content. It describes the steps in the integration of the DAK and review process of such complex documents as the standards for developing minimum variable product for the BornFyne v2.0 antenatal care feature. A key observation in this step relates to the absence of normative ANC guidelines at the health facility. Although health facilities have standard paper registers that are used to enter patient records, there are no guidelines for ANC to guide clinicians and ensure a systematic data collection process. This absence was observed as an important gap and was discussed with the national authorities. The team recommended using the existing DAK guidelines to contextualize and adapt a national normative guideline for ANC in Cameroon. This recommendation was welcomed by the Ministry of Public Health and documented as the next steps for the BornFyne team to include in the next phase of the implementation as the team moves towards developing the remaining DAK data elements into the BornFyne platform. The BornFyne team documented this as an important finding observed as a result of the integration and adaptation process of the DAK content into the BornFyne platform. Therefore, the DAK can help identify gaps in the way guidelines are managed or implemented within health systems at various levels. It can also facilitate the uptake of guidelines for countries that do not have existing guidelines and thus serves as a guide to contextualize and adapt a normative guideline for ANC for that country. Finally, it can help enhance the existing digital platform to align with standards that integrate ICD codes or other standards, as does the case for the BornFyne-PNMS v2.0.

The digital health ecosystem in Cameroon is still in an emerging phase and is not well-established. Nevertheless, there is an increasing demand for and penetration of digital health technologies in the country. As the BornFyne-PNMS was developed prior to the launch of the digital adaptation kit for ANC, the process of integrating the DAK into BornFyne-PNMS consisted more of expanding and updating the existing elements by reorganizing, reanalyzing, and remodeling the existing components to comply with the DAK elements. This meant executing the data elements aligned to the workflow and activities to generate activity codes to facilitate the process of interoperability.

This was a unique experience for the software engineers working on the BornFyne-PNMS project, as it was their first time reviewing and adapting comprehensive international guidelines that encompassed clinical and coded items, including decision support logic, and updating the BornFyne platform. The DAK has greatly facilitated the development and refinement of the BornFyne ANC module because the guidelines are clear and organized to ease the work of the software engineers. However, the software engineers faced some important challenges during the review stage in understanding medical terminology procedures in the DAK for the ANC context (processes, activities, and elements) and the prerequisites. The DAK’s detailed documentation and operational requirements for implementing the WHO recommendations in the digital systems guidelines helped guide the team in general. However, during the stakeholder discussions, we observed that the decision support logic, indicators, and performance metrics were important areas that could benefit from more stakeholder engagement to enhance further integration and operability, given the absence of normative ANC guidelines. It was necessary to take a step back to engage additional strategic stakeholders to advocate for adopting a normative guideline to enhance the quality of ANC and the performance of health providers.

Based on the lessons learned from implementing v1.0 and updating v2.0, the team, especially the software engineers, found the DAK data dictionary and decision support logic document to be an important tool for guiding and facilitating their work in terms of the structure of the document, the workflow processes, and content mapping to facilitate interoperability. In summary, the DAK was important for the planning phase as it helped identify important gaps in the absence of ANC guidelines at health facilities and the need to adopt a normative ANC guideline to enhance uptake, providers’ performance, and quality of ANC care delivered. Most importantly, it validates some of the challenges that are highlighted in Cameroon’s digital framework, which are in line with poor adherence to clinical protocol and quality of care. The software engineers on the team were much more comfortable with the scope and definition of data elements and the expectations and workflow processes in facilitating their work.

## 5 Recommendations and lessons for future research

Considering that we started at the health facility to explore normative guidelines for ANC before validating them at the Ministry of Public Health, this process highlighted the need for future research to determine whether such guidelines are available at the health facility and the Ministry of Public Health and whether they are actively used or simply shelved in health facilities across both urban and rural settings. In our case, this required extending validation efforts to regional and central levels. Therefore, it is important to ensure that normative guidelines are available at the Ministry of Public Health and that they are verified for their availability and active use at the point of care.

Tools like the Service Availability and Readiness Assessment (SARA) can provide initial insights into health facility readiness but may have limitations. For example, during our study, some facilities misidentified health facility registers as ANC guidelines, and without thorough validation by data collectors, such discrepancies may go unnoticed. It is crucial to adopt a bottom-up approach by involving health providers who deliver ANC, especially providers from rural health facilities, in content mapping. This helps identify any misidentification of ANC guidelines. In addition, their firsthand experience with daily challenges enables them to offer valuable insights and ensure practical alignment with guidelines. Considering that we targeted mostly rural health facilities, this observation and lessons learned provide insights into some of the challenges that hinder optimal quality of care for maternal health. Thus, it is an important lesson to engage ANC providers from rural and urban health facilities in mapping and design processes to ensure contextually appropriate solutions. Further research could explore how integrating additional DAK content domains (e.g., Post-natal Care (PNC) or Family Planning) into the system might enhance the delivery of integrated care within ANC and across other health areas.

## 6 Strengths and limitations

The participatory action research approach allowed us to engage relevant stakeholders involved in the development and implementation of the digital health project. This approach meant engaging multiple voices in the process, gathering relevant feedback, and increasing awareness of the BornFyne-PNMS digital platform to facilitate action and uptake (see Table 3). The study targets four districts, both English and French, and therefore provides feedback to inform the French context. It incorporates health providers from both public and private healthcare delivery sectors. Engaging key stakeholders ensured appropriate localization of the content. The study is focused on documenting the steps employed by the BornFyne project in updating and enhancing the DAK components within an existing platform that was initially designed before the launch of the DAK; thus, the approach may not be the same for an entirely new application. Another limitation was the absence of normative ANC guidelines to facilitate content adaptation; thus, we used health facility registers.

This document is limited to the assessment of the existing 40% elements and does not comprehensively report all the other components in the DAK that are yet to be integrated within the BornFyne. However, it provides a direction for the process and possible differences that are expected and can guide the next phase of completing the integration, contextualization, and adaptation of the DAK within the BornFyne-PNMS in Cameroon. Given the limited research and country experiences in integrating the DAK into digital systems, this approach and lessons learned can serve as a direction for other countries or teams planning to use the DAK to update an existing digital platform or to contextualize and adapt normative ANC guidelines.

## 7 Conclusion

The digital health ecosystem in Cameroon is still in an early maturity stage with an increasing demand for digital health technologies, especially in the area of reproductive, maternal, newborn, and child healthcare. The DAKs serve as operational and software-neutral mechanisms designed to convert WHO guidelines into standardized formats for ease of integration into digital systems across different countries. This article describes and documents the steps in the operationalization of the DAKs through the BornFyne-PNMS, highlighting the DAK as an important tool for guiding and facilitating software engineers in developing and integrating recommended guidelines into digital platforms and facilitates interoperability, going by the structure of the document, its workflow processes, and content mapping elements.

## Data Availability

The original contributions presented in the study are included in the article/Supplementary Material; further inquiries can be directed to the corresponding author.

## Abbreviations

ANC, antenatal care; DAK, digital adaptation kit; ICD, international classification of diseases; PNMS, prenatal management system; RMNCAH, reproductive maternal newborn child adolescent health; SMART, Standards-Based, Machine-Readable, Adaptive, Requirements-Based, and Testable; WHO, World Health Organization
