# Supplementary material for: Integrating WHO’s digital adaptation kit for antenatal care into BornFyne-PNMS: insights from Cameroon
Source: Front Pharmacol. 2025 Mar 26;16:1474999. doi: 10.3389/fphar.2025.1474999 (PMC11978650; doi:10.3389/fphar.2025.1474999)
Supplement: Supplementary file 1 [file Table1.pdf]

## Supplemental Table 1: Some DAK contents that are included in the BornFyne-PNMS v2.0 including the ICD codes.

**Registration**

| [ANC] Activity ID | [ANC] Data Element ID | ICD-11 Code | Data Element Label | Description and Definition | BornFyne V1 | BornFyne V2 |
|---|---|---|---|---|---|---|
| **ANC.A7. Create client record OR ANC.A8. Validate client details** | ANC.A7.DE1 | Not classifiable in ICD-11 | Unique identification | Unique identifier generated for new clients or a universal ID, if used in the country | P | P |
| **ANC.A7. Create client record OR ANC.A8. Validate client details** | ANC.A7.DE2 | Not classifiable in ICD-11 | First name | Client's first name | P | P |
| **ANC.A7. Create client record OR ANC.A8. Validate client details** | ANC.A7.DE3 | Not classifiable in ICD-11 | Last name | Client's family name or last name | P | P |
| **ANC.A7. Create client record OR ANC.A8. Validate client details** | ANC.A7.DE4 | Not classifiable in ICD-11 | Contact date | The date and time of the client's contact | A | P |
| **ANC.A7. Create client record OR ANC.A8. Validate client details** | ANC.A7.DE5 | Not classifiable in ICD-11 | Date of birth | The client's date of birth (DOB), if known | P | P |

| | | | | | | |
|---|---|---|---|---|---|---|
| **ANC.A7. Create client record**<br>**OR**<br>**ANC.A8. Validate client details** | ANC.A7.DE6 | XT7Q Early Adolescence<br>11th year of life through the end of the 14th<br><br>XT4T Middle Adolescence<br>15th year of life through the end of the 17th<br><br>XT9X Late Adolescence<br>18th year of life through the end of the 19th<br><br>XT15 Young Adult<br>20th year of life through the end of the 24th<br><br>XT6S Adult<br>25th year of life through the end of the 64th. | Age | Age (number of years) of the client based on date of birth | P | P |
| **ANC.A7. Create client record**<br>**OR**<br>**ANC.A8. Validate client details** | ANC.A7.DE7 | Not classifiable in ICD-11 | Address | Client's home address or address that the client is consenting to disclose | P | P |

| | | | | | | |
|---|---|---|---|---|---|---|
| **ANC.A7. Create client record<br>OR<br>ANC.A8. Validate client details** | ANC.A7.DE8 | Not classifiable in ICD-11 | Mobile phone number | Client's mobile phone number | P | P |
| **ANC.A7. Create client record<br>OR<br>ANC.A8. Validate client details** | ANC.A7.DE9 | Not classifiable in ICD-11 | Woman wants to receive reminders during pregnancy | Whether or not the woman wants to receive SMS or other messages regarding her ANC contacts and health status during pregnancy | P | P |
| **ANC.A7. Create client record<br>OR<br>ANC.A8. Validate client details** | ANC.A7.DE10 | Not classifiable in ICD-11 | Alternative contact's name | Name of an alternative contact, which could be next of kin (e.g. partner, mother, sibling); the alternative contact would be used in the case of an emergency situation | P | P |
| **ANC.A7. Create client record<br>OR<br>ANC.A8. Validate client details** | ANC.A7.DE11 | Not classifiable in ICD-11 | Alternative contact's phone number | Phone number of the alternative contact | A | P |
| **ANC.A7. Create client record<br>OR<br>ANC.A8. Validate client details** | ANC.A7.DE12 | Not classifiable in ICD-11 | ANC contact number | The ANC contact or visit number – recommended minimum is 8 contacts | A | P |
| **ANC.A7. Create client record<br>OR<br>ANC.A8. Validate client details** | **ANC.A7.DE13** | | **Co-habitants** | **Who does the client live with? It is important to know whether client lives with parents, other family members, a partner, friends, etc.** | A | P |

| | | | | | | |
|---|---|---|---|---|---|---|
| **ANC.A7. Create client record OR ANC.A8. Validate client details** | ANC.A7.DE14 | Not classifiable in ICD-11 | Parents | Client lives with parents | A | P |
| **ANC.A7. Create client record OR ANC.A8. Validate client details** | ANC.A7.DE15 | Not classifiable in ICD-11 | Siblings | Client only lives with siblings | A | P |
| **ANC.A7. Create client record OR ANC.A8. Validate client details** | ANC.A7.DE16 | Not classifiable in ICD-11 | Extended family | Client lives with extended family (e.g. aunt, cousin, grandparents) | A | P |
| **ANC.A7. Create client record OR ANC.A8. Validate client details** | ANC.A7.DE17 | Not classifiable in ICD-11 | Partner | Client lives with a romantic partner | A | P |
| **ANC.A7. Create client record OR ANC.A8. Validate client details** | ANC.A7.DE18 | Not classifiable in ICD-11 | Friend(s) | Client lives with friend(s) | A | P |
| **ANC.A7. Create client record OR ANC.A8. Validate client details** | ANC.A7.DE19 | Not classifiable in ICD-11 | No one | Client lives alone | A | P |

## Quick Checks

| [ANC] Activity ID | [ANC] Data Element ID | ICD-11 Code | Data Element Label | Description and Definition | BornFyne V1 | BornFyne V2 |
|---|---|---|---|---|---|---|
| **ANC.B4. Confirm pregnancy** | ANC.B4.DE1 | XT0S | Pregnancy confirmed | Pregnancy has been confirmed | A | P |
| **ANC.B5. Quick check** | ANC.B5.DE1 | | **Reason for coming to facility** | **Records the reason why the woman came to the health-care facility today** | A | P |
| **ANC.B5. Quick check** | ANC.B5.DE2 | Not classifiable in ICD-11 | First antenatal care contact | This is the woman's first ANC contact | A | P |
| **ANC.B5. Quick check** | ANC.B5.DE3 | Not classifiable in ICD-11 | Scheduled antenatal care contact | The woman is coming in for a scheduled ANC contact | A | P |
| **ANC.B5. Quick check** | ANC.B5.DE4 | Not classifiable in ICD-11 | Specific complaint related to antenatal care | The woman has a specific complaint that is outside of her ANC contact schedule | A | P |
| **ANC.B5. Quick check** | **ANC.B5.DE5** | | **Specific health concern(s)** | **If the woman came to the facility with a specific health concern, select the health concern(s) from the list** | A | P |
| **ANC.B5. Quick check** | ANC.B5.DE6 | MF3A | Abnormal vaginal discharge (physiological) (foul smelling) (curd like) | Woman has abnormal vaginal discharge | A | P |
| **ANC.B5. Quick check** | ANC.B5.DE7 | BA00.Z | Change in blood pressure – up (hypertension) | Woman has changes in blood pressure – increase in blood pressure | A | P |
| **ANC.B5. Quick check** | ANC.B5.DE8 | BA2Z | Change in blood pressure – down (hypotension) | Woman has changes in blood pressure – decrease in blood pressure | A | P |
| **ANC.B5. Quick check** | ANC.B5.DE9 | ME05.0 | Constipation | Woman is constipated | A | P |
| **ANC.B5. Quick check** | ANC.B5.DE10 | JA8D.Z | Contractions | Woman is having contractions | A | P |
| **ANC.B5. Quick check** | ANC.B5.DE11 | MD12 | Cough | Woman has a cough | A | P |
| **ANC.B5. Quick check** | ANC.B5.DE12 | ME05.1 | Diarrhoea | Woman has diarrhoea | A | P |

| | | | | | | |
|---|---|---|---|---|---|---|
| **ANC.B5. Quick check** | ANC.B5.DE13 | MB48.Z | Dizziness | Woman is dizzy | A | P |
| **ANC.B5. Quick check** | ANC.B5.DE14 | QE51.1 | Intimate partner violence | Woman has been subjected to violence by an intimate partner and/or in the home | A | P |
| **ANC.B5. Quick check** | ANC.B5.DE15 | JA86.Y | Fetal movements – none | Woman is experiencing no fetal movement | A | P |
| **ANC.B5. Quick check** | ANC.B5.DE16 | JA86.Y | Fetal movements – reduced/poor | Woman is experiencing reduced or poor fetal movement | A | P |
| **ANC.B5. Quick check** | ANC.B5.DE17 | MG26 | Fever | Woman has a fever (body temperature of 37 degrees Celsius or higher) | A | P |
| **ANC.B5. Quick check** | ANC.B5.DE18 | CA07.0 | Flu symptoms | Woman has flu-like symptoms | A | P |
| **ANC.B5. Quick check** | ANC.B5.DE19 | JA88.0 | Fluid loss (leaking) | Woman is experiencing fluid loss (leaking) | A | P |
| **ANC.B5. Quick check** | ANC.B5.DE20 | MG31.1 | Headache | Woman has a headache | A | P |
| **ANC.B5. Quick check** | ANC.B5.DE21 | MD95 | Heartburn | Woman is experiencing heartburn | A | P |
| **ANC.B5. Quick check** | ANC.B5.DE22 | ND56.Z | Injury | Woman has an injury | A | P |
| **ANC.B5. Quick check** | ANC.B5.DE23 | ME10.1 | Jaundice | Woman has yellowing skin or whites of the eyes | A | P |
| **ANC.B5. Quick check** | ANC.B5.DE24 | MB47.3 | Leg cramps | Woman has leg cramps | A | P |
| **ANC.B5. Quick check** | ANC.B5.DE25 | ME64.0 | Leg redness | Woman has redness in her leg(s) | A | P |
| **ANC.B5. Quick check** | ANC.B5.DE26 | 6B0Z | Mental health – Anxiety | Woman is experiencing anxiety | A | P |
| **ANC.B5. Quick check** | ANC.B5.DE27 | 6A7Z | Mental health – Depression | Woman is experiencing depression | A | P |

| | | | | | | |
|---|---|---|---|---|---|---|
| **ANC.B5. Quick check** | ANC.B5.DE28 | 6E8Z | Mental health – Other psychological symptoms | Woman has other psychological symptoms not described above | A | P |
| **ANC.B5. Quick check** | ANC.B5.DE29 | MD90.0 | Nausea | Woman is nauseous | A | P |
| **ANC.B5. Quick check** | ANC.B5.DE30 | MG29.0Y | Oedema | An excess of fluid in the tissues of the body. It causes excessive weight gain and swelling which puts on pressure. In pregnancy it is a common feature affecting the feet and ankles, but may also affect the hands, face and become generalized. | A | P |
| **ANC.B5. Quick check** | ANC.B5.DE31 | MD81.4 | Pain – Abdominal | Woman has abdominal pain | A | P |
| **ANC.B5. Quick check** | ANC.B5.DE32 | MF50.7 | Pain – During urination (dysuria) | Woman has pain during urination | A | P |
| **ANC.B5. Quick check** | ANC.B5.DE33 | FB56.4 | Pain – Leg | Woman has leg pain | A | P |
| **ANC.B5. Quick check** | ANC.B5.DE34 | ME84.2Z | Pain – Low back | Woman is experiencing back pain | A | P |
| **ANC.B5. Quick check** | ANC.B5.DE35 | MD81.11 | Pain – Pelvic | Woman is experiencing pelvic pain | A | P |
| **ANC.B5. Quick check** | ANC.B5.DE36 | JA65.7 | Pain – Extreme pelvic pain/cannot walk (symphysis pubis dysfunction) | Woman cannot walk due to extreme pelvic pain (symphysis pubis dysfunction) | A | P |
| **ANC.B5. Quick check** | ANC.B5.DE37 | MG3Z | Pain – Other | Woman is in pain, not described above | A | P |
| **ANC.B5. Quick check** | ANC.B5.DE38 | EC90.Z | Pruritus | Woman has severe itching of the skin | A | P |
| **ANC.B5. Quick check** | ANC.B5.DE39 | MD11.5 | Shortness of breath | Woman has shortness of breath | A | P |

| | | | | | | |
|---|---|---|---|---|---|---|
| **ANC.B5. Quick check** | ANC.B5.DE40 | MB22.7 | Tiredness | Woman is fatigued | A | P |
| **ANC.B5. Quick check** | ANC.B5.DE41 | JA41.Z | Vaginal bleeding | Woman is bleeding vaginally | A | P |
| **ANC.B5. Quick check** | ANC.B5.DE42 | MC1Y | Visual disturbance | Woman has disturbance in her vision including blurry vision, flashing lights, floaters, seeing stars or spots, having visual loss | A | P |
| **ANC.B5. Quick check** | ANC.B5.DE43 | MD90.1 | Vomiting | Woman is vomiting (not severely) | A | P |
| **ANC.B5. Quick check** | ANC.B5.DE44 | MG27 | Other bleeding | Woman is bleeding (not vaginally) | A | P |
| **ANC.B5. Quick check** | ANC.B5.DE45 | ME67 | Other skin disorder | Woman has other skin disorders not described above | A | P |
| **ANC.B5. Quick check** | ANC.B5.DE46 | PF2Z | Other types of violence | Woman has been subjected to other types of violence that is not domestic violence | A | P |
| **ANC.B5. Quick check** | ANC.B5.DE47 | Not classifiable in ICD-11 | Other complaint (specify) | Write in the other complaint not included in the list | A | P |
| **ANC.B5. Quick check** | **ANC.B5.DE48** | | **Danger signs** | **Before each contact, the health worker should check whether the woman has any of the danger signs listed here – if yes, she should refer to the hospital urgently; if no, she should continue to the normal contact** | A | P |
| **ANC.B5. Quick check** | ANC.B5.DE49 | Not classifiable in ICD-11 | No danger signs | No danger signs are present | A | P |
| **ANC.B5. Quick check** | ANC.B5.DE50 | JA41.Z | Bleeding vaginally | Woman is bleeding vaginally | A | P |
| **ANC.B5. Quick check** | ANC.B5.DE51 | ME64.1 | Central cyanosis | Woman has a bluish discolouration of the skin | A | P |
| **ANC.B5. Quick check** | ANC.B5.DE52 | 8A68.Z | Convulsing | Woman is convulsing | A | P |

| | | | | | | |
|---|---|---|---|---|---|---|
| **ANC.B5. Quick check** | ANC.B5.DE53 | MG26 | Fever | Woman has a fever (body temperature greater than or equal to 37°C) | A | P |
| **ANC.B5. Quick check** | ANC.B5.DE54 | JB0Z | Imminent delivery | Woman is about to give birth | A | P |
| **ANC.B5. Quick check** | ANC.B5.DE55 | JB0Z | Labour | Woman is in labour | A | P |
| **ANC.B5. Quick check** | ANC.B5.DE56 | MG48 | Looks very ill | Woman looks very ill | A | P |
| **ANC.B5. Quick check** | ANC.B5.DE57 | MG31.1 | Severe headache | Woman has a severe headache | A | P |
| **ANC.B5. Quick check** | ANC.B5.DE58 | MG3Z | Severe pain | Woman is in severe pain (not abdominal pain) | A | P |
| **ANC.B5. Quick check** | ANC.B5.DE59 | MD90.1 | Severe vomiting | Woman is vomiting severely | A | P |
| **ANC.B5. Quick check** | ANC.B5.DE60 | MD81.4 | Severe abdominal pain | Woman is in severe abdominal pain | A | P |
| **ANC.B5. Quick check** | ANC.B5.DE61 | MB20.1 | Unconscious | Woman is unconscious | A | P |
| **ANC.B5. Quick check** | ANC.B5.DE62 | MC1Y | Visual disturbance | Woman is visually disturbed | A | P |

## Profile and History

| [ANC] Activity ID | [ANC] Data element ID | ICD-11 Code | Data element label | Description and definition | BornFyne V1 | BornFyne V2 |
|---|---|---|---|---|---|---|
| **ANC.B6. Collect woman's profile and history** | **ANC.B6.DE1** | | **Highest level of education achieved** | **The highest level of schooling the woman has reached** | P | P |
| **ANC.B6. Collect woman's profile and history** | ANC.B6.DE2 | Not classifiable in ICD-11 | Does not know level of education | Woman does not know the level of education they have received | P | P |
| **ANC.B6. Collect woman's profile and history** | ANC.B6.DE3 | Not classifiable in ICD-11 | No education | Woman has received some primary education or no primary education | P | P |

| | | | | | | |
|---|---|---|---|---|---|---|
| **ANC.B6. Collect woman's profile and history** | ANC.B6.DE4 | Not classifiable in ICD-11 | Primary school | Woman has completed primary education | P | P |
| **ANC.B6. Collect woman's profile and history** | ANC.B6.DE5 | Not classifiable in ICD-11 | Secondary school | Woman has completed secondary education | P | P |
| **ANC.B6. Collect woman's profile and history** | ANC.B6.DE6 | Not classifiable in ICD-11 | Higher education | Woman has an undergraduate degree or higher | P | P |
| **ANC.B6. Collect woman's profile and history** | **ANC.B6.DE7** | | **Occupation** | **The woman's occupation (select all that apply)** | P | P |
| **ANC.B6. Collect woman's profile and history** | ANC.B6.DE8 | Not classifiable in ICD-11 | Student | Woman is pursuing education and is enrolled in school | P | P |
| **ANC.B6. Collect woman's profile and history** | ANC.B6.DE9 | QD80 | Unemployed | Woman is currently unemployed | P | P |
| **ANC.B6. Collect woman's profile and history** | ANC.B6.DE10 | Not classifiable in ICD-11 | Formal employment | Woman is formally employed at a formal organization (company, government, NGO, etc.) | P | P |
| **ANC.B6. Collect woman's profile and history** | ANC.B6.DE11 | Not classifiable in ICD-11 | Employment that puts woman at increased risk for HIV (e.g. sex worker) | Woman's employment puts her at increased risk for HIV | P | P |
| **ANC.B6. Collect woman's profile and history** | ANC.B6.DE12 | Not classifiable in ICD-11 | Informal employment (other) | Woman is currently engaged in informal forms of employment | P | P |
| **ANC.B6. Collect woman's profile and history** | ANC.B6.DE13 | Not classifiable in ICD-11 | Other (specify) | The above descriptions do not capture the woman's occupation – specify in text here | P | P |
| **ANC.B6. Collect woman's profile and history** | **ANC.B6.DE14** | | **Gestational age** | | A | P |
| **ANC.B6. Collect woman's profile and history** | ANC.B6.DE14 | Not classifiable in ICD-11 | Last menstrual period (LMP) date | The woman's last menstrual period (LMP) date; this is defined as the first day of her most recent period | A | P |
| **ANC.B6. Collect woman's profile and history** | ANC.B6.DE15 | Not classifiable in ICD-11 | Ultrasound done | Whether or not the woman has had an ultrasound scan done at any point in this pregnancy | A | P |
| **ANC.B6. Collect woman's profile and** | ANC.B6.DE16 | Not classifiable in ICD-11 | Ultrasound date | Date that the ultrasound was done | A | P |

| | | | | | | |
|---|---|---|---|---|---|---|
| **history** | | | | | | |
| **ANC.B6. Collect woman's profile and history** | ANC.B6.DE17 | XT3X | Gestational age | Gestational age in weeks and/or days depending on the source of gestational age | A | P |
| **ANC.B6. Collect woman's profile and history** | **ANC.B6.DE18** | | **Source of gestational age** | **Gestational age can be calculated multiple ways – this data element describes where the gestational age above has been calculated from** | A | P |
| **ANC.B6. Collect woman's profile and history** | ANC.B6.DE19 | Not classifiable in ICD-11 | Last menstrual period (LMP) | Gestational age (GA) calculated from LMP | P | P |
| **ANC.B6. Collect woman's profile and history** | ANC.B6.DE20 | Not classifiable in ICD-11 | Ultrasound | Woman's gestational age today calculated using ultrasound GA and ultrasound date | A | P |
| **ANC.B6. Collect woman's profile and history** | ANC.B6.DE21 | Not classifiable in ICD-11 | Symphysis fundal height (SFH) or abdominal palpation | If LMP is unknown and either ultrasound wasn't done or it wasn't done early enough, then show the option for health worker to enter GA in weeks based on symphysis fundal height (SFH) or abdominal palpation | A | P |
| **ANC.B6. Collect woman's profile and history** | ANC.B6.DE22 | Not classifiable in ICD-11 | Expected date of delivery (EDD) | Expected date of delivery based on gestational age | A | P |
| **ANC.B6. Collect woman's profile and history** | **ANC.B6.DE23** | | **Number of previous pregnancies** | **This calculates the total number of all previous pregnancies (i.e. not including this current pregnancy); this is done for easier obstetric history calculations** | P | P |
| **ANC.B6. Collect woman's profile and history** | ANC.B6.DE24 | Not classifiable in ICD-11 | Number of pregnancies (gravida) | Total number of times the woman has been pregnant (including this pregnancy); also referred to as gravida | P | P |
| **ANC.B6. Collect woman's profile and history** | ANC.B6.DE25 | Not classifiable in ICD-11 | Number of miscarriages and/or abortions | Total number of pregnancies lost/ended due to miscarriages and/or abortions before 22 weeks / 5 months | A | P |
| **ANC.B6. Collect woman's profile and history** | ANC.B6.DE26 | Not classifiable in ICD-11 | Number of live births | Total number of live births after 22 weeks | P | P |

| | | | | | | |
|---|---|---|---|---|---|---|
| **ANC.B6. Collect woman's profile and history** | **ANC.B6.DE27** | | **Whether last live birth was preterm** | **Was the last live birth preterm? Whether the last live birth was preterm (i.e. less than 37 weeks gestation at the time of delivery)** | A | P |
| **ANC.B6. Collect woman's profile and history** | ANC.B6.DE28 | Not classifiable in ICD-11 | Last live birth was preterm | Yes, last live birth was preterm | A | P |
| **ANC.B6. Collect woman's profile and history** | ANC.B6.DE29 | Not classifiable in ICD-11 | Last live birth was not preterm | No, last live birth was not preterm | A | P |
| **ANC.B6. Collect woman's profile and history** | ANC.B6.DE30 | Not classifiable in ICD-11 | Don't know if last live birth was preterm | Don't know if last live birth was preterm | A | P |
| **ANC.B6. Collect woman's profile and history** | ANC.B6.DE31 | Not classifiable in ICD-11 | Number of stillbirths | Total number of stillbirths after 22 weeks | A | P |
| **ANC.B6. Collect woman's profile and history** | ANC.B6.DE32 | Not classifiable in ICD-11 | Parity | Calculated parity is the total number of live and stillbirths | A | P |
| **ANC.B6. Collect woman's profile and history** | ANC.B6.DE33 | Not classifiable in ICD-11 | Number of caesarian sections | Total number of caesarean sections | P | P |
| **ANC.B6. Collect woman's profile and history** | **ANC.B6.DE34** | | **Past pregnancy complications** | **Mark whether the woman has had any complications or problems in any previous pregnancy** | P(Antenatal History text) | P |
| **ANC.B6. Collect woman's profile and history** | ANC.B6.DE35 | Not classifiable in ICD-11 | No past pregnancy complications | No known past pregnancy problems | A | P |
| **ANC.B6. Collect woman's profile and history** | ANC.B6.DE36 | Not classifiable in ICD-11 | Does not know of any past pregnancy complications | Don't know if there were any problems during past pregnancies | A | P |

| | | | | | | |
|---|---|---|---|---|---|---|
| **ANC.B6. Collect woman's profile and history** | ANC.B6.DE37 | JA24.Z | Pre-eclampsia | A condition specific to pregnancy, arising after the 20th week of gestation, characterized by hypertension and proteinuria. Oedema may also be present, but is no longer considered a cardinal sign because it is present to some extent in most pregnancies. If not controlled, pre-eclampsia will lead to eclampsia which is characterized by fits, followed by coma, and has a high mortality rate. | P | P |
| **ANC.B6. Collect woman's profile and history** | ANC.B6.DE38 | JA25.3 | Eclampsia | A condition peculiar to pregnancy or a newly delivered woman, characterized by fits followed coma. The woman usually has hypertension and proteinuria. The fits may occur in the antepartum, intrapartum or early postpartum periods. | A | P |
| **ANC.B6. Collect woman's profile and history** | ANC.B6.DE39 | QE10 | Alcohol use | Alcohol intake | A | P |
| **ANC.B6. Collect woman's profile and history** | ANC.B6.DE40 | KD3B.Z | Baby died within 24 hours of birth | The woman's baby died within 24 hours of childbirth | A | P |
| **ANC.B6. Collect woman's profile and history** | ANC.B6.DE41 | 8A68.Z | Convulsions | Woman had convulsions during her past pregnancy | A | P |
| **ANC.B6. Collect woman's profile and history** | ANC.B6.DE42 | JB21 | Forceps | Woman required forceps delivery | A | P |
| **ANC.B6. Collect woman's profile and history** | ANC.B6.DE43 | JA63.2 | Gestational diabetes mellitus | Hyperglycaemia first detected at any time during pregnancy | A | P |
| **ANC.B6. Collect woman's profile and history** | ANC.B6.DE44 | JA43.1 | Heavy bleeding (during or after delivery) | Woman was bleeding heavily during or after giving birth during previous pregnancy/pregnancies | A | P |
| **ANC.B6. Collect woman's profile and history** | ANC.B6.DE45 | JA86.5 | Macrosomia | Fetus large for gestational age | A | P |

| | | | | | | |
|---|---|---|---|---|---|---|
| **ANC.B6. Collect woman's profile and history** | ANC.B6.DE46 | JB09.2 | Perineal tear (3rd or 4th degree) | Woman experienced 3rd or 4th degree perineal tear | A | P |
| **ANC.B6. Collect woman's profile and history** | ANC.B6.DE47 | QE11.8 | Substance use | Illicit drug use (e.g. cannabis, amphetamines, prescription stimulants, opioids, opiates, ecstasy, cocaine) | A | P |
| **ANC.B6. Collect woman's profile and history** | ANC.B6.DE48 | QE13 | Tobacco use | Use of tobacco products, in any form | A | P |
| **ANC.B6. Collect woman's profile and history** | ANC.B6.DE49 | JB21 | Vacuum delivery | Woman required vacuum delivery | A | P |
| **ANC.B6. Collect woman's profile and history** | ANC.B6.DE50 | Not classifiable in ICD-11 | Other past pregnancy problems (specify) | Woman experienced other past pregnancy problems not described above | A | P |
| **ANC.B6. Collect woman's profile and history** | **ANC.B6.DE51** | | **Substance use during past pregnancy specification** | **If selected substance use, specify and indicate what type of substances used during past pregnancies** | A | P |
| **ANC.B6. Collect woman's profile and history** | ANC.B6.DE53 | QE11.3 | Cocaine | Cocaine hydrochloride | A | P |
| **ANC.B6. Collect woman's profile and history** | ANC.B6.DE54 | QE11.8 | Injectable drugs | Drugs that are received through intravenous injections, e.g. heroin | A | P |
| **ANC.B6. Collect woman's profile and history** | ANC.B6.DE55 | QE11.1 | Marijuana | Formally known as cannabis; other names include, weed, hash, mary jane | A | P |
| **ANC.B6. Collect woman's profile and history** | ANC.B6.DE56 | Not classifiablein ICD-11 | Other illicit substance use (specify) | Other illicit substances not described above, e.g. amphetamines, ecstasy, other opiates | A | P |
| **ANC.B6. Collect woman's profile and history** | **ANC.B6.DE57** | | **Allergies** | **Does the woman have any allergies?** | A | P |
| **ANC.B6. Collect woman's profile and history** | ANC.B6.DE58 | Not classifiable in ICD-11 | No known allergies | Woman does not have any known allergies | A | P |
| **ANC.B6. Collect woman's profile and history** | ANC.B6.DE59 | XM79J1 | Albendazole | Allergy to albendazole | A | P |
| **ANC.B6. Collect woman's profile and history** | ANC.B6.DE60 | XM9DS4 | Aluminium hydroxide | Allergy to aluminium hydroxide | A | P |

| | | | | | | |
|---|---|---|---|---|---|---|
| **ANC.B6. Collect woman's profile and history** | ANC.B6.DE61 | XM8KC2 | Calcium | Allergy to calcium | A | P |
| **ANC.B6. Collect woman's profile and history** | ANC.B6.DE62 | XM7CX5 | Chamomile | Allergy to chamomile | A | P |
| **ANC.B6. Collect woman's profile and history** | ANC.B6.DE63 | XM7R82 | Folic acid | Allergy to folic acid | A | P |
| **ANC.B6. Collect woman's profile and history** | ANC.B6.DE64 | XM1TN8 | Ginger | Allergy to ginger | A | P |
| **ANC.B6. Collect woman's profile and history** | ANC.B6.DE65 | XM8Z42 | Iron | Allergy to iron | A | P |
| **ANC.B6. Collect woman's profile and history** | ANC.B6.DE66 | XM7V20 | Magnesium carbonate | Allergy to magensium carbonate | A | P |
| **ANC.B6. Collect woman's profile and history** | ANC.B6.DE67 | XM90Z4 | Malaria medication (sulfadoxine-pyrimethamine) | Allergy to malaria medication (sulfadoxine-pyrimethamine) | A | P |
| **ANC.B6. Collect woman's profile and history** | ANC.B6.DE68 | XM3GX0 | Mebendazole | Allergy to mebendazole | A | P |
| **ANC.B6. Collect woman's profile and history** | ANC.B6.DE69 | XM7Q57 | Penicillin | Allergy to penicillin | A | P |
| **ANC.B6. Collect woman's profile and history** | ANC.B6.DE70 | XM63K0 | PrEP tenofovir disoproxil fumarate (TDF) | Allergy to PrEP tenofovir disoproxil fumarate (TDF) | A | P |
| **ANC.B6. Collect woman's profile and history** | ANC.B6.DE71 | Not classifiable in ICD-11 | Other allergies (specify) | Woman has other allergies not listed here; please specify | A | P |
| **ANC.B6. Collect woman's profile and history** | **ANC.B6.DE72** | | **Past surgeries** | **Has the woman had any prior surgeries?** | P(Medical History) | P |
| **ANC.B6. Collect woman's profile and history** | ANC.B6.DE73 | Not classifiable in ICD-11 | No known past surgeries | Woman has not had past surgeries | A | P |
| **ANC.B6. Collect woman's profile and history** | ANC.B6.DE74 | Not classifiable in ICD-11 | Does not know of any past surgeries | Woman does not know if she has had past surgeries | A | P |
| **ANC.B6. Collect woman's profile and history** | ANC.B6.DE75 | QC48.Y | Dilation and curettage | Woman had dilation and curettage procedure | A | P |

| | | | | | | |
|---|---|---|---|---|---|---|
| **ANC.B6. Collect woman's profile and history** | ANC.B6.DE76 | QC48.Y | Myomectomy | Removal of fibroids (myomectomy) | A | P |
| **ANC.B6. Collect woman's profile and history** | ANC.B6.DE77 | QC48.Y | Removal of ovarian cysts | Woman has had ovarian cysts removed | A | P |
| **ANC.B6. Collect woman's profile and history** | ANC.B6.DE78 | QC48.Y | Oophorectomy | Removal of ovary (oophorectomy) | A | P |
| **ANC.B6. Collect woman's profile and history** | ANC.B6.DE79 | QC48.Y | Salpingectomy | Removal of the fallopian tube (salpingectomy) | A | P |
| **ANC.B6. Collect woman's profile and history** | ANC.B6.DE80 | QC48.Y | Cervical cone | Partial removal of the cervix (cervical cone) | A | P |
| **ANC.B6. Collect woman's profile and history** | ANC.B6.DE81 | QC48.Y | Other gynecological procedures (specify) | Write in the other gynecological procedures not included in the list | A | P |
| **ANC.B6. Collect woman's profile and history** | ANC.B6.DE82 | Not classifiable in ICD-11 | Other surgeries (specify) | Write in the other surgeries not included in the list | A | P |
| **ANC.B6. Collect woman's profile and history** | **ANC.B6.DE83** | | **Existing chronic health conditions** | **Does the woman have any current chronic health conditions or problems?** | A | P |
| **ANC.B6. Collect woman's profile and history** | ANC.B6.DE84 | Not classifiable in ICD-11 | No chronic or past health conditions | Woman does not have chronic diseases or any past health conditions | A | P |
| **ANC.B6. Collect woman's profile and history** | ANC.B6.DE85 | Not classifiable in ICD-11 | Don't know if there are any chronic or past health conditions | Woman does not know whether she has any chronic diseases or past health conditions | A | P |
| **ANC.B6. Collect woman's profile and history** | ANC.B6.DE86 | 4A4Z | Autoimmune disease | Woman has an autoimmune disease | A | P |
| **ANC.B6. Collect woman's profile and history** | ANC.B6.DE87 | 3C0Z | Blood disorder (e.g. sickle cell, anaemia, thalassemia) | Woman has a blood disorder | A | P |
| **ANC.B6. Collect woman's profile and history** | ANC.B6.DE88 | 2C7Z | Cancer – gynaecological | Woman has gynaecological cancer | A | P |
| **ANC.B6. Collect woman's profile and history** | ANC.B6.DE89 | Not classifiable in ICD-11 | Cancer – other site (specify) | Woman has other type of cancer | A | P |
| **ANC.B6. Collect woman's profile and history** | ANC.B6.DE90 | JA63.2 | Diabetes arising in pregnancy (gestational diabetes) | Woman has diabetes arising in pregnancy (gestational diabetes) | A | P |

| | | | | | | |
|---|---|---|---|---|---|---|
| **ANC.B6. Collect woman's profile and history** | ANC.B6.DE91 | JA63.Z | Diabetes, other or unspecified | Woman has diabetes of other or unspecified type | A | P |
| **ANC.B6. Collect woman's profile and history** | ANC.B6.DE92 | JA63.0 | Diabetes, pre-existing type 1 | Woman has pre-existing type 1 diabetes mellitus | A | P |
| **ANC.B6. Collect woman's profile and history** | ANC.B6.DE93 | JA63.1 | Diabetes, pre-existing type 2 | Woman has pre-existing type 2 diabetes mellitus | A | P |
| **ANC.B6. Collect woman's profile and history** | ANC.B6.DE94 | 8A6Z | Epilepsy | Woman has epilepsy | A | P |
| **ANC.B6. Collect woman's profile and history** | ANC.B6.DE95 | 1C62.Z | HIV positive | Woman is HIV positive | P | P |
| **ANC.B6. Collect woman's profile and history** | ANC.B6.DE96 | BA00.Z | Hypertension | High blood pressure | P | P |
| **ANC.B6. Collect woman's profile and history** | ANC.B6.DE97 | GC2Z | Kidney disease | Woman has chronic kidney disease | A | P |
| **ANC.B6. Collect woman's profile and history** | ANC.B6.DE98 | Not classifiable in ICD-11 | Other (specify) | Write in the other health conditions not included in the list | A | P |
| **ANC.B6. Collect woman's profile and history** | ANC.B6.DE99 | Not classifiable in ICD-11 | HIV diagnosis date | If woman knows she's HIV+, record the date of her diagnosis | A | P |
| **ANC.B6. Collect woman's profile and history** | **ANC.B6.DE100** | | **Tetanus toxoid-containing vaccine (TTCV) immunization history** | **The woman's history of receiving tetanus toxoid-containing vaccine (TTCV)** | A | P |
| **ANC.B6. Collect woman's profile and history** | ANC.B6.DE101 | Not classifiable in ICD-11 | Fully immunized | Pregnant woman is fully protected against tetanus (i.e. has received 6 TTCV doses in childhood/adolescence, or 5 doses if first vaccinated after 1 year of age/during adolescence/adulthood, including during previous pregnancies), and no further vaccination is needed. | A | P |

| | | | | | | |
|---|---|---|---|---|---|---|
| **ANC.B6. Collect woman's profile and history** | ANC.B6.DE102 | QC00.5 | Under-immunized | If the pregnant woman has received 1–4 doses of TTCV in the past, administer one dose of TTCV before delivery. | A | P |
| **ANC.B6. Collect woman's profile and history** | ANC.B6.DE103 | QC00.5 | No doses | TTCV has never been provided: no dose, or zero doses. Woman should receive at least 2 TTCV doses as early as possible, with an interval of 4 weeks between the doses. Administer the 2nd dose at least 2 weeks before birth to allow for adequate immune response. | A | P |
| **ANC.B6. Collect woman's profile and history** | ANC.B6.DE104 | Not classifiable in ICD-11 | Unknown | Woman does not know whether or not they have received any doses of TTCV. She should receive at least 2 TTCV doses as early as possible, with an interval of 4 weeks between the doses. Administer the 2nd dose at least 2 weeks before birth to allow for adequate immune response. | A | P |
| **ANC.B6. Collect woman's profile and history** | **ANC.B6.DE105** | | **Flu immunization history** | **Whether or not this year's seasonal flu vaccine has been provided** | A | P |
| **ANC.B6. Collect woman's profile and history** | ANC.B6.DE106 | Not classifiable in ICD-11 | Fully immunized | Flu immunization provided | A | P |
| **ANC.B6. Collect woman's profile and history** | ANC.B6.DE107 | QC01.8 | No doses | The woman has not been immunized with this year's seasonal flu vaccine, thus she is under-immunized for the flu this year | A | P |
| **ANC.B6. Collect woman's profile and history** | ANC.B6.DE108 | Not classifiable in ICD-11 | Unknown | Woman does not know whether or not they have received the seasonal flu vaccine | A | P |
| **ANC.B6. Collect woman's profile and history** | **ANC.B6.DE109** | | **Current medications** | **Select all of the medications the woman is currently taking** | A | P |

| | | | | | | |
|---|---|---|---|---|---|---|
| **ANC.B6. Collect woman's profile and history** | ANC.B6.DE110 | Not classifiable in ICD-11 | No medications | The woman is currently not on any medications | A | P |
| **ANC.B6. Collect woman's profile and history** | ANC.B6.DE111 | Not classifiable in ICD-11 | Does not know of any current medications | The woman does not know if she is on any medications | A | P |
| **ANC.B6. Collect woman's profile and history** | ANC.B6.DE112 | XM4S22 | Analgesic | Analgesic, or painkiller, medication | A | P |
| **ANC.B6. Collect woman's profile and history** | ANC.B6.DE113 | XM37S3 | Antacids | Antacids | A | P |
| **ANC.B6. Collect woman's profile and history** | ANC.B6.DE114 | XM80V1 | Anti-convulsive | Anti-convulsive medication | A | P |
| **ANC.B6. Collect woman's profile and history** | ANC.B6.DE115 | XM8S35 | Anti-diabetic | Anti-diabetic medication | A | P |
| **ANC.B6. Collect woman's profile and history** | ANC.B6.DE116 | XM4EC0 | Antihelmintic | Antihelmintic, or anti-parasitic, medication | A | P |
| **ANC.B6. Collect woman's profile and history** | ANC.B6.DE117 | XM2PT6 | Anti-hypertensive | Anti-hypertensive medication | A | P |
| **ANC.B6. Collect woman's profile and history** | ANC.B6.DE118 | XM1914 | Anti-malarials | Anti-malarial medication | A | P |
| **ANC.B6. Collect woman's profile and history** | ANC.B6.DE119 | XM63K0 | Antiretrovirals (ARVs) | Antiretrovirals (ARVs) | A | P |
| **ANC.B6. Collect woman's profile and history** | ANC.B6.DE120 | XM05R0 | Antitussive | Antitussive, or cough suppressant, medication | A | P |
| **ANC.B6. Collect woman's profile and history** | ANC.B6.DE121 | XM63K0 | Antivirals | Antiviral medication | A | P |
| **ANC.B6. Collect woman's profile and history** | ANC.B6.DE122 | XM4G06 | Aspirin | Aspirin | A | P |
| **ANC.B6. Collect woman's profile and history** | ANC.B6.DE123 | XM8MA0 | Asthma | Asthma medication | A | P |
| **ANC.B6. Collect woman's profile and history** | ANC.B6.DE124 | XM8KC2 | Calcium | Calcium supplements | A | P |

| | | | | | | |
|---|---|---|---|---|---|---|
| **ANC.B6. Collect woman's profile and history** | ANC.B6.DE125 | XM22Y8 | Co-trimoxazole | Co-trimoxazole medication | A | P |
| **ANC.B6. Collect woman's profile and history** | ANC.B6.DE126 | XM77K6 | Doxylamine | Doxylamine | A | P |
| **ANC.B6. Collect woman's profile and history** | ANC.B6.DE127 | XM7R82 | Folic acid | Folic acid supplements | P | P |
| **ANC.B6. Collect woman's profile and history** | ANC.B6.DE128 | XM20E6 | Hematinic | Hematinic supplements | A | P |
| **ANC.B6. Collect woman's profile and history** | ANC.B6.DE129 | XM38V9 | Hemorrhoidal medication | Hemorrhoidal medication | A | P |
| **ANC.B6. Collect woman's profile and history** | ANC.B6.DE130 | XM8Z42 | Iron | Iron supplements | P | P |
| **ANC.B6. Collect woman's profile and history** | ANC.B6.DE131 | XM5TD2 | Magnesium | Magnesium supplements | A | P |
| **ANC.B6. Collect woman's profile and history** | ANC.B6.DE132 | XM3XX3 | Metoclopramide | Metoclopramide | A | P |
| **ANC.B6. Collect woman's profile and history** | ANC.B6.DE133 | XM7XY8 | Multivitamin | Multivitamin supplements | A | P |
| **ANC.B6. Collect woman's profile and history** | ANC.B6.DE134 | XM63K0 | Oral pre-exposure prophylaxis (PrEP) for HIV | Oral pre-exposure prophylaxis (PrEP) | A | P |
| **ANC.B6. Collect woman's profile and history** | ANC.B6.DE135 | XM7SQ4 | Other antibiotics | Other antibiotics not listed above | A | P |
| **ANC.B6. Collect woman's profile and history** | ANC.B6.DE136 | XM5685 | Thyroid medication | Medication to address thyroid issues | A | P |
| **ANC.B6. Collect woman's profile and history** | ANC.B6.DE137 | XM17W6 | Vitamin A | Vitamin A supplements | A | P |
| **ANC.B6. Collect woman's profile and history** | ANC.B6.DE138 | Not classifiable in ICD-11 | Other medications (specify) | Other medications or supplements that are not listed above | A | P |
| **ANC.B6. Collect woman's profile and history** | **ANC.B6.DE139** | | **Daily caffeine intake** | **Assesses whether the woman consumes more than 300 mg of caffeine per day** | A | P |

| | | | | | | |
|---|---|---|---|---|---|---|
| **ANC.B6. Collect woman's profile and history** | ANC.B6.DE140 | XM9KW2 | More than 2 cups of coffee (brewed, filtered, instant or espresso) | More than 2 cups of coffee (brewed, filtered, instant or espresso) | A | P |
| **ANC.B6. Collect woman's profile and history** | ANC.B6.DE141 | XM0NG8 | More than 4 cups of tea | More than 4 cups of tea | A | P |
| **ANC.B6. Collect woman's profile and history** | ANC.B6.DE142 | XM0NG8 | More than 12 bars (50 g) of chocolate | More than 12 bars (50 g) of chocolate | A | P |
| **ANC.B6. Collect woman's profile and history** | ANC.B6.DE143 | XM0NG8 | More than one can of soda or energy drink | More than one can of soda or energy drink | A | P |
| **ANC.B6. Collect woman's profile and history** | ANC.B6.DE144 | Not classifiable in ICD-11 | None of the above daily caffeine intake | None of the above daily caffeine intake applies to the women | A | P |
| **ANC.B6. Collect woman's profile and history** | **ANC.B6.DE145** | | **Current alcohol and/or other substance use** | **Whether or not the woman currently consumes any alcohol or substances** | A | P |
| **ANC.B6. Collect woman's profile and history** | ANC.B6.DE146 | Not classifiable in ICD-11 | Clinical enquiry for alcohol and other substance use done | Whether or not the health worker has performed a clinical enquiry into alcohol and substance use | A | P |
| **ANC.B6. Collect woman's profile and history** | ANC.B6.DE147 | Not classifiable in ICD-11 | No alcohol and/or substances | The woman currently does not consume alcohol and/or any substances | A | P |
| **ANC.B6. Collect woman's profile and history** | ANC.B6.DE148 | QE10 | Alcohol | Woman currently consumes alcohol | A | P |
| **ANC.B6. Collect woman's profile and history** | ANC.B6.DE149 | QE11.3 | Cocaine | Woman currently uses cocaine | A | P |
| **ANC.B6. Collect woman's profile and history** | ANC.B6.DE150 | QE11.8 | Injectable drugs | Woman currently uses injectable drugs | A | P |
| **ANC.B6. Collect woman's profile and history** | ANC.B6.DE151 | QE11.1 | Marijuana | Woman currently uses marijuana | A | P |
| **ANC.B6. Collect woman's profile and history** | ANC.B6.DE152 | Not classifiable in ICD-11 | Other substance use (specify) | Woman uses other substances not listed above | A | P |
| **ANC.B6. Collect woman's profile and history** | **ANC.B6.DE153** | **QE13** | **Tobacco use** | **Whether the woman uses tobacco products** | A | P |

| | | | | | | |
|---|---|---|---|---|---|---|
| **ANC.B6. Collect woman's profile and history** | ANC.B6.DE154 | QC46 | Recently quit tobacco products | Whether the woman has recently quit using any tobacco products | A | P |
| **ANC.B6. Collect woman's profile and history** | ANC.B6.DE155 | QD70.5 | Exposure to second-hand smoke | Anyone in the household smokes tobacco products – whether the woman is exposed to second-hand smoke from anyone in her household | A | P |
| **ANC.B6. Collect woman's profile and history** | **ANC.B6.DE156** | | **Partner HIV status (reported)** | **The HIV status of the woman's partner** | A | P |
| **ANC.B6. Collect woman's profile and history** | ANC.B6.DE157 | QC90.6 | HIV positive | Woman's partner is HIV positive | A | P |
| **ANC.B6. Collect woman's profile and history** | ANC.B6.DE158 | Not classifiable in ICD-11 | HIV negative | Woman's partner is HIV negative | A | P |
| **ANC.B6. Collect woman's profile and history** | ANC.B6.DE159 | Not classifiable in ICD-11 | Inconclusive | Don't know HIV status – woman does not know partner's HIV status | A | P |
| **ANC.B6. Collect woman's profile and history** | ANC.B6.DE160 | QA21.6Y | Contraceptive use of male condoms | Whether or not the woman (and her partner) use male condoms during sex | P | P |
| **ANC.B6. Collect woman's profile and history** | ANC.B6.DE161 | QA21.6Y | Contraceptive use of female condoms | Whether or not the woman (and her partner) use female condoms during sex | A | P |

**Physical Exams**

| [ANC] Activity ID | [ANC] Data Element ID | ICD-11 Code | Data Element Label | Description and Definition | BornFyne V1 | BornFyne V2 |
|---|---|---|---|---|---|---|
| **ANC.B8. Conduct physical exam** | | | **Height and weight** | | P | P |
| **ANC.B8. Conduct physical exam** | **ANC.B8.DE1** | Not classifiable in ICD-11 | Height | The woman's current height in centimetres | P | P |
| **ANC.B8. Conduct physical exam** | ANC.B8.DE2 | Not classifiable in ICD-11 | Pre-gestational weight | The woman's pre-gestational weight in kilograms | A | P |

| | | | | | | |
|---|---|---|---|---|---|---|
| **ANC.B8. Conduct physical exam** | ANC.B8.DE3 | Not classifiable in ICD-11 | Current weight | The woman's current weight in kilograms | P | P |
| **ANC.B8. Conduct physical exam** | ANC.B8.DE4 | Not classifiable in ICD-11 | BMI | Body mass index (BMI): calculated by taking weight in kg divided by the squared height in meters, i.e. kg/(m^2) | A | P |
| **ANC.B8. Conduct physical exam** | **ANC.B8.DE5** | | **Weight category** | **Weight category is calculated from BMI** | A | P |
| **ANC.B8. Conduct physical exam** | ANC.B8.DE6 | 5B54 | Underweight | Woman has a BMI that is defined as underweight (less than 18.5) | A | P |
| **ANC.B8. Conduct physical exam** | ANC.B8.DE7 | Not classifiable in ICD-11 | Normal weight | Woman has a BMI that is defined as normal (BMI is 18.5 – less than 25) | A | P |
| **ANC.B8. Conduct physical exam** | ANC.B8.DE8 | 5B80.01 | Overweight | Woman has a BMI that is defined as overweight (25 – less than 30) | A | P |
| **ANC.B8. Conduct physical exam** | ANC.B8.DE9 | 5B81.Z | Obese | Woman has a BMI that is defined as obese (over 30) | A | P |
| **ANC.B8. Conduct physical exam** | **ANC.B8.DE10** | | **Expected weight gain** | **Expected weight gain during pregnancy is based on the woman's weight category** | A | P |
| **ANC.B8. Conduct physical exam** | ANC.B8.DE11 | Not classifiable in ICD-11 | 5–9 kg | Woman is expected to gain 5–9 kg during pregnancy | A | P |
| **ANC.B8. Conduct physical exam** | ANC.B8.DE12 | Not classifiable in ICD-11 | 7–11.5 kg | Woman is expected to gain 7–11.5 kg during pregnancy | A | P |
| **ANC.B8. Conduct physical exam** | ANC.B8.DE13 | Not classifiable in ICD-11 | 11.5–16 kg | Woman is expected to gain 11.5–16 kg during pregnancy | A | P |
| **ANC.B8. Conduct physical exam** | ANC.B8.DE14 | Not classifiable in ICD-11 | 12.5–18 kg | Woman is expected to gain 12.5–18 kg during pregnancy | A | P |
| **ANC.B8. Conduct physical exam** | ANC.B8.DE15 | Not classifiable in ICD-11 | Average weight gain per week | Average weight gain per week since last contact (kg); this would only display starting at the 2nd contact | A | P |
| **ANC.B8. Conduct physical exam** | ANC.B8.DE16 | Not classifiable in ICD-11 | Total weight gain (kg) | Total weight gain in pregnancy so far (kg) | A | P |

| | | | | | | |
|---|---|---|---|---|---|---|
| **ANC.B8. Conduct physical exam** | | | **Blood pressure** | | P | P |
| **ANC.B8. Conduct physical exam** | ANC.B8.DE17 | Not classifiable in ICD-11 | Systolic blood pressure | Systolic blood pressure (SBP) in mmHg | P | P |
| **ANC.B8. Conduct physical exam** | ANC.B8.DE19 | Not classifiable in ICD-11 | Diastolic blood pressure | Diastolic blood pressure (DBP) in mmHg | P | P |
| **ANC.B8. Conduct physical exam** | ANC.B8.DE20 | Not classifiable in ICD-11 | Repeat systolic blood pressure | Repeat measurement of the woman's systolic blood pressure in mmHg after 10–15 minutes rest | A | P |
| **ANC.B8. Conduct physical exam** | ANC.B8.DE21 | Not classifiable in ICD-11 | Repeat diastolic blood pressure | Repeat measurement of the woman's diastolic blood pressure in mmHg after 10–15 minutes rest | A | P |
| **ANC.B8. Conduct physical exam** | ANC.B8.DE22 | QB14 | Blood pressure cannot be taken | If the woman's blood pressure cannot be taken, this should be indicated here; otherwise, blood pressure should be measured | A | P |
| **ANC.B8. Conduct physical exam** | **ANC.B8.DE23** | | **Reason blood pressure cannot be taken** | **The reason why the health worker is unable to record the woman's BP during this contact visit** | A | P |
| **ANC.B8. Conduct physical exam** | ANC.B8.DE24 | QB14 | BP cuff (sphygmomanometer) not available | Blood pressure cuff is not available | A | P |
| **ANC.B8. Conduct physical exam** | ANC.B8.DE25 | QB14 | BP cuff (sphygmomanometer) is broken | Blood pressure cuff is broken | A | P |
| **ANC.B8. Conduct physical exam** | ANC.B8.DE26 | Not classifiable in ICD-11 | Other (specify) | Other reason blood pressure cannot be taken | A | P |
| **ANC.B8. Conduct physical exam** | **ANC.B8.DE27** | | **Symptoms of severe pre-eclampsia** | **Check if the woman has any of the following symptoms of severe pre-eclampsia** | A | P |
| **ANC.B8. Conduct physical exam** | ANC.B8.DE28 | Not classifiable in ICD-11 | No symptoms of severe pre-eclampsia | Woman does not have symptoms of severe pre-eclampsia | A | P |
| **ANC.B8. Conduct physical exam** | ANC.B8.DE29 | MG31.1 | Severe headache | Woman has a severe headache | A | P |

| | | | | | | |
|---|---|---|---|---|---|---|
| **ANC.B8. Conduct physical exam** | ANC.B8.DE30 | MC1Y | Visual disturbance | Woman has disturbance in her vision | A | P |
| **ANC.B8. Conduct physical exam** | ANC.B8.DE31 | MD81.10 | Epigastric pain | Woman is experiencing epigastric pain | A | P |
| **ANC.B8. Conduct physical exam** | ANC.B8.DE32 | MB48.Z | Dizziness | Woman is dizzy | A | P |
| **ANC.B8. Conduct physical exam** | ANC.B8.DE33 | MD90.1 | Vomiting | Woman is vomiting | A | P |
| | | | **Temperature, pulse and pallor** | | A | P |
| **ANC.B8. Conduct physical exam** | ANC.B8.DE34 | Not classifiable in ICD-11 | Body temperature | The woman's body temperature in degrees Celsius (°C) (first reading) | P | P |
| **ANC.B8. Conduct physical exam** | ANC.B8.DE35 | Not classifiable in ICD-11 | Second body temperature | The woman's body temperature in degrees Celsius (°C) (second reading) | A | P |
| **ANC.B8. Conduct physical exam** | ANC.B8.DE36 | Not classifiable in ICD-11 | Pulse rate | The woman's pulse rate in beats per minute (bpm) | P | P |
| **ANC.B8. Conduct physical exam** | ANC.B8.DE37 | Not classifiable in ICD-11 | Second pulse rate | The woman's second pulse rate in beats per minute (bpm) | A | P |
| **ANC.B8. Conduct physical exam** | ANC.B8.DE38 | ME64.2 | Pallor present | Whether or not the woman has pallor | A | P |

*Note: The first five columns are from the DAK. A=Absent: P=Present (No data element of ICD code in BornFyne-PNMS version 1.0, updated version 2.0 has data element ID and ICD codes).*
