# Supplementary material for: Integrating WHO’s digital adaptation kit for antenatal care into BornFyne-PNMS: insights from Cameroon
Source: Front Pharmacol. 2025 Mar 26;16:1474999. doi: 10.3389/fphar.2025.1474999 (PMC11978650; doi:10.3389/fphar.2025.1474999)
Supplement: Supplementary file 2 [file Image4.pdf]

**Supplemental Figure 4: Output on integration of WHO DAK content into BornFyne-PNMS content Client details with corresponding ICD codes from WHO DAK**

| | A | B | C | D | E |
|---|---|---|---|---|---|
| 1 | Column1 | Column2 | Column3 | Column4 | Column5 |
| 2 | Activity ID Activity Name | Data Element ID | Data Element Name | Description and Defintion | ICD CODE |
| 3 | ANC.A4 Gather client details | ANC.A4.DE12 | Co-habitants | Who the client lives with (e.g. parents [in cases of adolescents], partner, extended family, siblings, friend[s], | |
| 4 | ANC.A4 Gather client details | ANC.A4.DE11 | Alternativecontact's phone number | Phone number for the alternative contact | |
| 5 | ANC.A4 Gather client details | ANC.A4.DE10 | Alternative contact's name | Name of an alternative contact, which could be next of kin (e.g. partner, mother, sibling); the alternative co | |
| 6 | ANC.A4 Gather client details | ANC.A4.DE9 | Woman wants to receivereminders during pregnancy? (optional) | Whether or not the woman wants to receive SMS or other messages regarding her ANC contacts and health s | |
| 7 | ANC.A4 Gather client details | ANC.A4.DE8 | Mobile phone number (optional) | Client's mobile phone number | |
| 8 | ANC.A4 Gather client details | ANC.A4.DE7 | Address | Client's homeaddress or address that the client is consenting to disclose | |
| 9 | ANC.A4 Gather client details | ANC.A4.DE6 | Age | Age (number of years) of the client based on the DOB | |
| 10 | ANC.A4 Gather client details | ANC.A4.DE5 | Date of birth (DOB) | Client's DOB, if known | |
| 11 | ANC.A4 Gather client details | ANC.A4.DE4 | Contact date | The dateand timeof the client's contact | |
| 12 | ANC.A4 Gather client details | ANC.A4.DE3 | Last name | Client's family name or last name | |
| 13 | ANC.A4 Gather client details | ANC.A4.DE2 | First name | Client's first name | |
| 14 | ANC.A4 Gather client details | ANC.A4.DE1 | Unique Identification | Unique identifier generated for new clients or a universal ID, if used in the country | |
| 15 | ANC.A7 Create client record | ANC.A7.DE19 | No one | Client livesalone | Not classifiable in ICD-11 |
| 16 | ANC.A7 Create client record | ANC.A7.DE13 | Co-habitants | Who does the client live with? It is important to know whether client lives with parents, other family memb | |
| 17 | ANC.A7 Create client record | ANC.A7.DE18 | Friend(s) | Client lives with friend(s) | Not classifiable in ICD-11 |
| 18 | ANC.A7 Create client record | ANC.A7.DE12 | ANC contactnumber | The ANC contact or visit number – recommended minimum is 8 contacts | Not classifiable in ICD-11 |
| 19 | ANC.A7 Create client record | ANC.A7.DE17 | Partner | Client lives with a romantic partner | Not classifiable in ICD-11 |
| 20 | ANC.A7 Create client record | ANC. A7.DE11 | Alternative contact's phone number | Phone number of the alternative contact | Not classifiable in ICD-11 |
| 21 | ANC. A7Create client record | ANC. A7.DE16 | Extended family | Client lives with extended lamily (e.g. aunt, cousin, grandparents) | Not classifiable in ICD-11 |
| 22 | ANC.A7 Create client record | ANC.A7.DE10 | Alternative contact's name | Name of an alternativecontact, which could be next of kin (e.g. partner, mother, sibling); the alternative co | Not classifiable in ICD-11 |
| 23 | ANC.A7 Create client record | ANC.A7.DE9 | Woman wants to receive reminders during pregnancy | Whether or not the woman wants to receive SMS or other messages regarding her ANC contacts and health s | Not classifiable in ICD-11 |
| 24 | ANC.A7 Create client record | ANC.A7. DE15 | Siblings | Client only lives with siblings | Not classifiable in ICD-11 |
| 25 | ANC.A7 Create client record | ANC.A7.DE8 | Mobile phone number | Client's mobile phone number | Not classifiable in ICD-11 |
| 26 | ANC.A7 Create client record | ANC.A7.DE14 | Parents | Client lives with parents | Not classifiable in ICD-11 |
| 27 | ANC.A7 Create client record | ANC.A7.DE7 | Address | Client's home address or address that the client is consenting to disclose | Not classifiable in ICD-11 |
| 28 | ANC.A7 Create client record | ANC.A7.DE6 | Age | Age (number of years) of the client based on date of birth | XT6S Adult 25th year of life through the end of the 64th. |
| 29 | ANC.A7 Create client record | ANC.A7.DE5 | Date of birth | The client's date of birth (DOB), if known | Not classifiable in ICD-11 |
| 30 | ANC.A7 Create client record | ANC.A7.DE4 | Contact date | The dateand time of the client's contact | Not classifiable in ICD-11 |
| 31 | ANC.A7 Create client record | ANC.A7.DE3 | Last name | Client's family name or last name | Not classifiable in ICD-11 |
| 32 | ANC.A7 Create client record | ANC.A7.DE2 | First name | Client's first name | Not classifiable in ICD-11 |
