# Supplementary material for: Integrating WHO’s digital adaptation kit for antenatal care into BornFyne-PNMS: insights from Cameroon
Source: Front Pharmacol. 2025 Mar 26;16:1474999. doi: 10.3389/fphar.2025.1474999 (PMC11978650; doi:10.3389/fphar.2025.1474999)
Supplement: Supplementary file 3 [file Image2.pdf]

Supplemental Figure 2: DAK Elements in BornFyne-PNMS version 1.0 versus updated version 2.0 ANC Profile and History

1	[ANC] Activity ID	[ANC] Data element ID	ICD-11 Code	Data element label	Description and definition	BornFyne V1	BornFyne V2
3	ANC.B6. Collect woman's profile and history	ANC.B6.DE2	Not classifiable in ICD-11	Does not know level of education	Woman does not know the level of education they have received	P	P
4	ANC.B6. Collect woman's profile and history	ANC.B6.DE3	Not classifiable in ICD-11	No education	Woman has received some primary education or no primary education	P	P
5	ANC.B6. Collect woman's profile and history	ANC.B6.DE4	Not classifiable in ICD-11	Primary school	Woman has completed primary education	P	P
6	ANC.B6. Collect woman's profile and history	ANC.B6.DE5	Not classifiable in ICD-11	Secondary school	Woman has completed secondary education	P	P
7	ANC.B6. Collect woman's profile and history	ANC.B6.DE6	Not classifiable in ICD-11	Higher education	Woman has an undergraduate degree or higher	P	P
8	**ANC.B6. Collect woman's profile and history**	**ANC.B6.DE7**		**Occupation**	**The woman's occupation (select all that apply)**	P	P
9	ANC.B6. Collect woman's profile and history	ANC.B6.DE8	Not classifiable in ICD-11	Student	Woman is pursuing education and is enrolled in school	P	P
10	ANC.B6. Collect woman's profile and history	ANC.B6.DE9	QD80	Unemployed	Woman is currently unemployed	P	P
11	ANC.B6. Collect woman's profile and history	ANC.B6.DE10	Not classifiable in ICD-11	Formal employment	Woman is formally employed at a formal organization (company, government, NGO, etc.)	P	P
12	ANC.B6. Collect woman's profile and history	ANC.B6.DE11	Not classifiable in ICD-11	Employment that puts woman at increased risk for HIV (e.g. sex worker)	Woman's employment puts her at increased risk for HIV	P	P
13	ANC.B6. Collect woman's profile and history	ANC.B6.DE12	Not classifiable in ICD-11	Informal employment (other)	Woman is currently engaged in informal forms of employment	P	P
14	ANC.B6. Collect woman's profile and history	ANC.B6.DE13	Not classifiable in ICD-11	Other (specify)	The above descriptions do not capture the woman's occupation – specify in text here	P	P
15	**ANC.B6. Collect woman's profile and history**	**ANC.B6.DE14**		**Gestational age**		A	P
16	ANC.B6. Collect woman's profile and history	ANC.B6.DE14	Not classifiable in ICD-11	Last menstrual period (LMP) date	The woman's last menstrual period (LMP) date; this is defined as the first day of her most recent period	A	P
17	ANC.B6. Collect woman's profile and history	ANC.B6.DE15	Not classifiable in ICD-11	Ultrasound done	Whether or not the woman has had an ultrasound scan done at any point in this pregnancy	A	P
18	ANC.B6. Collect woman's profile and history	ANC.B6.DE16	Not classifiable in ICD-11	Ultrasound date	Date that the ultrasound was done	A	P
19	ANC.B6. Collect woman's profile and history	ANC.B6.DE17	XT3X	Gestational age	Gestational age in weeks and/or days depending on the source of gestational age	A	P
20	**ANC.B6. Collect woman's profile and history**	**ANC.B6.DE18**		**Source of gestational age**	**Gestational age can be calculated multiple ways – this data element describes where the gestational age above has been calculated from**	A	P

The first five columns are from the DAK. A=Absent: P=Present (No data element of ICD code in BornFyne-PNMS version 1.0, updated version 2.0 has data element ID and ICD codes).
