# Supplementary material for: Integrating WHO’s digital adaptation kit for antenatal care into BornFyne-PNMS: insights from Cameroon
Source: Front Pharmacol. 2025 Mar 26;16:1474999. doi: 10.3389/fphar.2025.1474999 (PMC11978650; doi:10.3389/fphar.2025.1474999)
Supplement: Supplementary file 4 [file Image3.pdf]

Supplemental Figure 3: Output on integration of WHO DAK content into BornFyne-PNMS content on quick checks with corresponding ICD codes from WHO DAK

	Column1	Column2	Column3	Column4	Column5
36	ANC.B5 Quick check	ANC.B5.DE5-DE47	Specific health concern(s)	If the woman came to the facility with a specific health concern, select the health concern(s) from the list	
37	ANC.B5 Quick check	ANC.B5.DE53	Fever	Woman has a fever (body temperature greater than or equal to 37°C)	MG26
38	ANC.B5 Quick check	ANC.B5.DE1-DE54	Reason for coming to facility	Records the reason why the woman came to the health-care facility today	
39	ANC.B5 Quick check	ANC.B5.DE52	Convulsing	Woman is convulsing	8A68.Z
40	ANC.B5 Quick check	ANC.B5.DE8	Change in blood pressure – down (hypotension)	Woman has changes in blood pressure – decrease in blood pressure	BA2Z
41	ANC.B5 Quick check	ANC.B5.DE51	Central cyanosis	Woman has a bluish discolouration of the skin	ME64.1
42	ANC.B5 Quick check	ANC.B5.DE7	Change in blood pressure – up (hypertension)	Woman has changes in blood pressure – increase in blood pressure	BA00.Z
43	ANC.B5 Quick check	ANC.B5.DE50	Bleeding vaginally	Woman is bleeding vaginally	JA41.Z
44	ANC.B5 Quick check	ANC.B5.DE6	Abnormal vaginal discharge (physiological) (foul smelling) (curd like)	Woman has abnormal vaginal discharge	MF3A
45	ANC.B5 Quick check	ANC.B5.DE49	No danger signs	No danger signs are present	Not classifiable in ICD-11
46	ANC.B5 Quick check	ANC.B5.DE5	Specific health concern(s)	If the woman came to the facility with a specific health concern, select the health concern(s) from the list	
47	ANC.B5 Quick check	ANC.B5.DE48	Danger signs	Before each contact, the health worker should check whether the woman has any of the danger signs listed	
48	ANC.B5 Quick check	ANC.B5.DE4	Specific complaint related to antenatal care	The woman has a specific complaint that is outside of her ANC contact schedule	Not classifiable in ICD-11
49	ANC.B5 Quick check	ANC.B5.DE42	Visual disturbance	Woman has disturbance in her vision including blurry vision, flashing lights, floaters, seeing stars or spots, h	MC1Y
50	ANC.B5 Quick check	ANC.B5.DE47	Other complaint (specify)	Write in the other complaint not included in the list	Not classifiable in ICD-11
51	ANC.B5 Quick check	ANC.B5.DE3	Scheduled antenatal care contact	The woman is coming in for a scheduled ANC contact	Not classifiable in ICD-11
52	ANC.B5 Quick check	ANC.B5.DE41	Vaginal bleeding	Woman is bleeding vaginally	JA41.Z
53	ANC.B5 Quick check	ANC.B5.DE46	Other types of violence	Woman has been subjected to other types of violence that is not domestic violence	PF2Z
54	ANC.B5 Quick check	ANC.B5.DE2	First antenatal care contact	This is the woman's first ANC contact	Not classifiable in ICD-11
55	ANC.B5 Quick check	ANC.B5.DE40	Tiredness	Woman is fatigued	MB22.7
56	ANC.B5 Quick check	ANC.B5.DE45	Other skin disorder	Woman has other skin disorders not described above	ME67
57	ANC.B5 Quick check	ANC.B5.DE1	Reason for coming to facility	Records the reason why the woman came to the health-care facility today	
58	ANC.B5 Quick check	ANC.B5.DE39	Shortness of breath	Woman has shortness of breath	MD11.5
59	ANC.B5 Quick check	ANC.B5.DE38	Pruritus	Woman has severe itching of the skin	EC90.Z
60	ANC.B5 Quick check	ANC.B5.DE44	Other bleeding	Woman is bleeding (not vaginally)	MG27
61	ANC.B5 Quick check	ANC.B5.DE37	Pain – Other	Woman is in pain, not described above	MG3Z
62	ANC.B5 Quick check	ANC.B5.DE43	Vomiting	Woman is vomiting (not severely)	MD90.1
63	ANC.B5 Quick check	ANC.B5.DE36	Pain – Extreme pelvic pain / cannot walk (symphysis pubis dysfunctio	Woman cannot walk due to extreme pelvic pain (symphysis pubis dysfunction)	JA65.7
64	ANC.B5 Quick check	ANC.B5.DE35	Pain – Pelvic	Woman is experiencing pelvic pain	MD81.11
65	ANC.B5 Quick check	ANC.B5.DE34	Pain – Low back	Woman is experiencing back pain	ME84.2Z
66	ANC.B5 Quick check	ANC.B5.DE33	Pain – Leg	Woman has leg pain	FB56.4
67	ANC.B5 Quick check	ANC.B5.DE32	Pain – During urination (dysuria)	Woman has pain during urination	MF50.7
[illegible]	[illegible]	[illegible]	[illegible]	[illegible]	[illegible]
