# Supplementary material for: Integrating WHO’s digital adaptation kit for antenatal care into BornFyne-PNMS: insights from Cameroon
Source: Front Pharmacol. 2025 Mar 26;16:1474999. doi: 10.3389/fphar.2025.1474999 (PMC11978650; doi:10.3389/fphar.2025.1474999)
Supplement: Supplementary file 5 [file Image1.pdf]

Supplemental Figure 1: DAK Elements in BornFyne-PNMS version 1.0 versus updated version

2.0 Physical Exam

[ANC] Activity ID	[ANC] Data Element ID	ICD-11 Code	Data Element Label	Description and Definition	BornFyne V1	BornFyne V2
ANC.B8. Conduct physical exam			Height and weight		P	P
ANC.B8. Conduct physical exam	ANC.B8.DE1	Not classifiable in ICD-11	Height	The woman's current height in centimetres	P	P
ANC.B8. Conduct physical exam	ANC.B8.DE2	Not classifiable in ICD-11	Pre-gestational weight	The woman's pre-gestational weight in kilograms	A	P
ANC.B8. Conduct physical exam	ANC.B8.DE3	Not classifiable in ICD-11	Current weight	The woman's current weight in kilograms	P	P
ANC.B8. Conduct physical exam	ANC.B8.DE4	Not classifiable in ICD-11	BMI	Body mass index (BMI): calculated by taking weight in kg divided by the squared height in meters, i.e. kg/(m^2)	A	P
ANC.B8. Conduct physical exam	ANC.B8.DE5		Weight category	Weight category is calculated from BMI	A	P
ANC.B8. Conduct physical exam	ANC.B8.DE6	5B54	Underweight	Woman has a BMI that is defined as underweight (less than 18.5)	A	P
ANC.B8. Conduct physical exam	ANC.B8.DE7	Not classifiable in ICD-11	Normal weight	Woman has a BMI that is defined as normal (BMI is 18.5 – less than 25)	A	P
ANC.B8. Conduct physical exam	ANC.B8.DE8	5B80.01	Overweight	Woman has a BMI that is defined as overweight (25 – less than 30)	A	P
ANC.B8. Conduct physical exam	ANC.B8.DE9	5B81.Z	Obese	Woman has a BMI that is defined as obese (over 30)	A	P
ANC.B8. Conduct physical exam	ANC.B8.DE10		Expected weight gain	Expected weight gain during pregnancy is based on the woman's weight category	A	P
ANC.B8. Conduct physical exam	ANC.B8.DE11	Not classifiable in ICD-11	5–9 kg	Woman is expected to gain 5–9 kg during pregnancy	A	P
ANC.B8. Conduct physical exam	ANC.B8.DE12	Not classifiable in ICD-11	7–11.5 kg	Woman is expected to gain 7–11.5 kg during pregnancy	A	P
ANC.B8. Conduct physical exam	ANC.B8.DE13	Not classifiable in ICD-11	11.5–16 kg	Woman is expected to gain 11.5–16 kg during pregnancy	A	P
ANC.B8. Conduct physical exam	ANC.B8.DE14	Not classifiable in ICD-11	12.5–18 kg	Woman is expected to gain 12.5–18 kg during pregnancy	A	P
ANC.B8. Conduct physical exam	ANC.B8.DE15	Not classifiable in ICD-11	Average weight gain per week	Average weight gain per week since last contact (kg); this would only display starting at the 2nd contact	A	P
ANC.B8. Conduct physical exam	ANC.B8.DE16	Not classifiable in ICD-11	Total weight gain (kg)	Total weight gain in pregnancy so far (kg)	A	P
ANC.B8. Conduct physical exam			Blood pressure		P	P
ANC.B8. Conduct physical exam	ANC.B8.DE17	Not classifiable in ICD-11	Systolic blood pressure	Systolic blood pressure (SBP) in mmHg	P	P

ANC.A. Registration | ANC.B5 Quick check | ANC.B6 Profile | ANC.B8 Physical exam | ANC.B9 Lab tests & imaging | +

Ready Accessibility: Investigate

The first five columns are from the DAK. A=Absent: P=Present (No data element of ICD code in BornFyne-PNMS version 1.0, updated version 2.0 has data element ID and ICD codes).
